# Multiproxy study of 7500-year-old wooden sickles from the Lakeshore Village of La Marmotta, Italy

**DOI:** 10.1038/s41598-022-18597-8

**Published:** 2022-09-02

**Authors:** Niccolò Mazzucco, Mario Mineo, Daniele Arobba, Rosanna Caramiello, Laura Caruso Fermé, Bernard Gassin, Denis Guilbeau, Juan José Ibáñez, Lionello F. Morandi, Millán Mozota, Fiona Pichon, Marta Portillo, Maxime Rageot, Gerard Remolins, Mauro Rottoli, Juan F. Gibaja

**Affiliations:** 1 Dipartimento di Civiltà e Forme del Sapere, Università di Pisa, Pisa, Italy; 2 Museo Delle Civiltà (MuCiv), Rome, Italy; 3 Museo Archeologico del Finale, Istituto Internazionale di Studi Liguri, Bordighera, Italy; 4 Dipartimento di Scienza Della Vita e Biologia dei Sistemi, Università di Torino, Turin, Italy; 5 Instituto Patagónico de Ciencias Sociales y Humanas (IPCSH-CONICET), Puerto Madryn, Argentina; 6 Travaux et Recherches Archéologiques sur les Cultures, Les Espaces et les Sociétés (TRACES) UMR 5608 CNRS, Université Toulouse Jean Jaurès, Toulouse, France; 7 Ministry of Culture/Archéologie des Sociétés Méditerranéennes (ASM) UMR 5140 CNRS, Université Paul Valéry Montpellier III, Montpellier, France; 8 Archaeology of Social Dynamics, Institución Milá y Fontanals de Investigación en Humanidades (IMF-CSIC), Barcelona, Spain; 9 Competence Center Archaeometry Baden-Württemberg, Eberhard-Karls-Universität Tübingen, Tübingen, Germany; 10 Institución Milá y Fontanals de Investigación en Humanidades (IMF-CSIC), Barcelona, Spain; 11 Department of Pre- and Protohistory, University of Tübingen and Ludwig Maximilian University of Munich, Munich, Germany; 12 Università di Bari, CNR-ISPC, Valencia, Spain; 13 Laboratorio di Archeobiologia dei Musei Civici di Como, Como, Italy; 14 Escuela Española de Historia y Arqueología en Roma, EEHAR-CSIC, Roma, Italy

**Keywords:** Archaeology, Social anthropology, Palaeoecology, Cultural evolution

## Abstract

The lakeshore site of La Marmotta is one of the most important Early Neolithic sites of Mediterranean Europe. The site is famous for the exceptional preservation of organic materials, including numerous wooden artefacts related to navigation, agriculture, textile production, and basketry. This article presents interdisciplinary research on three of the most complete and well-preserved sickles recovered from the site, yet unpublished. All the components of the tools are analysed: the stone inserts, the wooden haft and the adhesive substances used to fix the stones inside the haft. Our innovative methodology combines use-wear and microtexture analysis of stone tools through confocal microscopy, taxonomical and technological analysis of wood, gas chromatography–mass spectrometry analysis of the adhesive substances, and pollen, non-pollen palynomorphs, and phytolith analysis of the remains incorporated within the adhesive. This multiproxy approach provides a significant insight into the life of these tools, from their production to their use and abandonment, providing evidence of the species of harvested plants and the conditions of the field during the harvesting.

## Introduction

Harvesting techniques are a fundamental element of both past and modern farming technologies. Specific tools were used for harvesting cereals even prior to cereal domestication. At the submerged site of Ohalo II, located on the southwest shore of the Sea of Galilee in Israel, during the Late Glacial Maximum (ca. 23,000 cal BP), flaked stone tools, probably hafted into a sickle handle, were used to gather barley and wheat^1,2^. Direct evidence of the use of a sickle for harvesting wild cereals in the Early Natufian was found at the site of Wadi Hammeh 27, in northwest Jordan, where a complete antler sickle, dated back to 14,000 cal BP, was recovered^3^. Fragments of sickle hafts carved into ungulate bones have also been found at the Natufian sites of Kebara Cave and El Wad (Mount Carmel, Israel), Hayonim Cave (western Galilee, Israel), and Azraq 18 (eastern Jordan)^4–7^. During the course of Pre-Pottery Neolithic (PPNA and PPNB), the presence of sickle-blades is one of the most characterising elements of the archaeological assemblages^8–10^, and their frequency in lithic collections increases considerably between 11,000 BP and 9000 cal BP^11^. Sickles underwent several changes, gradually shifting from straight to curved hafts during the Middle and Late PPNB^12^, as shown by the remains of a sickle found at Tell Halula in the middle Euphrates valley (Syria) (*ca.* 9500–9000 cal BP)^13^.

As from *ca*. 8600 cal BP onwards^14^ farming began to spread into the Mediterranean basin, Neolithic groups brought domesticated crops to new regions together with the set of technologies and knowledge necessary to cultivate, harvest, store, process and consume them. Harvesting tools were definitely one fundamental part of this package, an important functional category, but probably also charged with aesthetic and identity values^15,16^. In the Central and Western Mediterranean, the first Neolithic occupations were associated with Impresso-Cardial Ware^17–20^. Those early communities brought the so-called Neolithic package; consisting of plants and animals domesticated in the Near East, along with specific architectural technologies, ceramic styles, ornaments and flaked and polished tool technologies.

Unfortunately, in most of the archaeological contexts perishable materials are not preserved and little information on the morphology, size and use of sickles is therefore available. Stone inserts are often all that remain of those complex instruments^21–24^. Research on stone tools indicates that adaptations were made to the harvesting toolkit during the migration process. While in the Near East, sickle-blade production was characterised by an increasing technological investment in their manufacture, from the Natufian to PN, early Mediterranean sickle inserts were, in general, largely produced through a relatively simple and not particularly specialised flaking process. Throughout the migration process, harvesting technologies were adapted to different economic, technological and social conditions^25^. Despite that, given the paucity of archaeological evidence, information about Neolithic harvesting practises is still fragmentary, largely relying only on data from stone tools and archaeobotanical records^11,25^.

For this reason, an invaluable source of information is represented by those sites characterised by a good preservation of organic remains (i.e. submerged, lakeshore or lake dwelling sites). Through the study of their exceptional archaeological record, our perception of Neolithic societies can change profoundly. This is the case of a lakeshore site, excavated between 1992 and 2006 in the Lake of Bracciano, near Rome (Italy): La Marmotta^26–28^. Among the extraordinary materials recovered from the excavations (i.e. five dugout canoes and numerous objects related to navigation, basketry and all kinds of wooden crafts), 52 wooden sickles were brought to light. These finds, including both complete and fragmentary specimens, represent the largest collection of Neolithic sickles in the entire Mediterranean Basin. Until now, La Marmotta sickles had never been published except for a few drawings and photos, and their potential remained unexploited^28^. However, harvesting tools can inform us about several aspects of Neolithic farming. The adoption of a specific harvesting method is a response to technical, economic and cultural determinants. For example, harvesting techniques are often adapted to the type of cultivated plants and the products sought (e.g., whole plants, whole ears only, individual grains, leaves only, etc.), but other factors influence as well, for example the production scale and the size of the harvested fields. In addition, age- and gender-specific aspects can also play an important role, as for example tool’s weight and shape may be suited for specific ages or sexes^29^. This knowledge, which was culturally transmitted from generation to generation, enabled Neolithic groups to successfully colonise new territories^30–32^.

In this research, we address the study of the three most complete and extraordinarily well-preserved La Marmotta sickles. The three sickles display considerable typological variations between each other in the shape of the handle and the size of the cutting edge. They are therefore representative of the diversity of sickles recovered at the site.

This study has been conducted through an innovative interdisciplinary approach. All the components of the tools are analysed: the stone inserts, the wooden haft and the adhesive substances used to fix the stones inside the haft. The current study followed an integrated analytical approach combining: (1) use-wear and microtexture analysis of stone tools through confocal microscopy, (2) taxonomical and technological analysis of wood, (3) gas chromatography–mass spectrometry (GC–MS) analysis of the adhesive substances, and (4) pollen, non-pollen palynomorphs (NPP), and phytolith analysis of the remains incorporated within the adhesive. Such an integrated approach allowed us not only to reconstruct the manufacture, use and management of the tools, but also to characterise more precisely the type of cultivated cereals and the conditions of the field during the harvesting.

## Materials and methods

### Materials

#### The archaeological excavations

**Figure 1:**
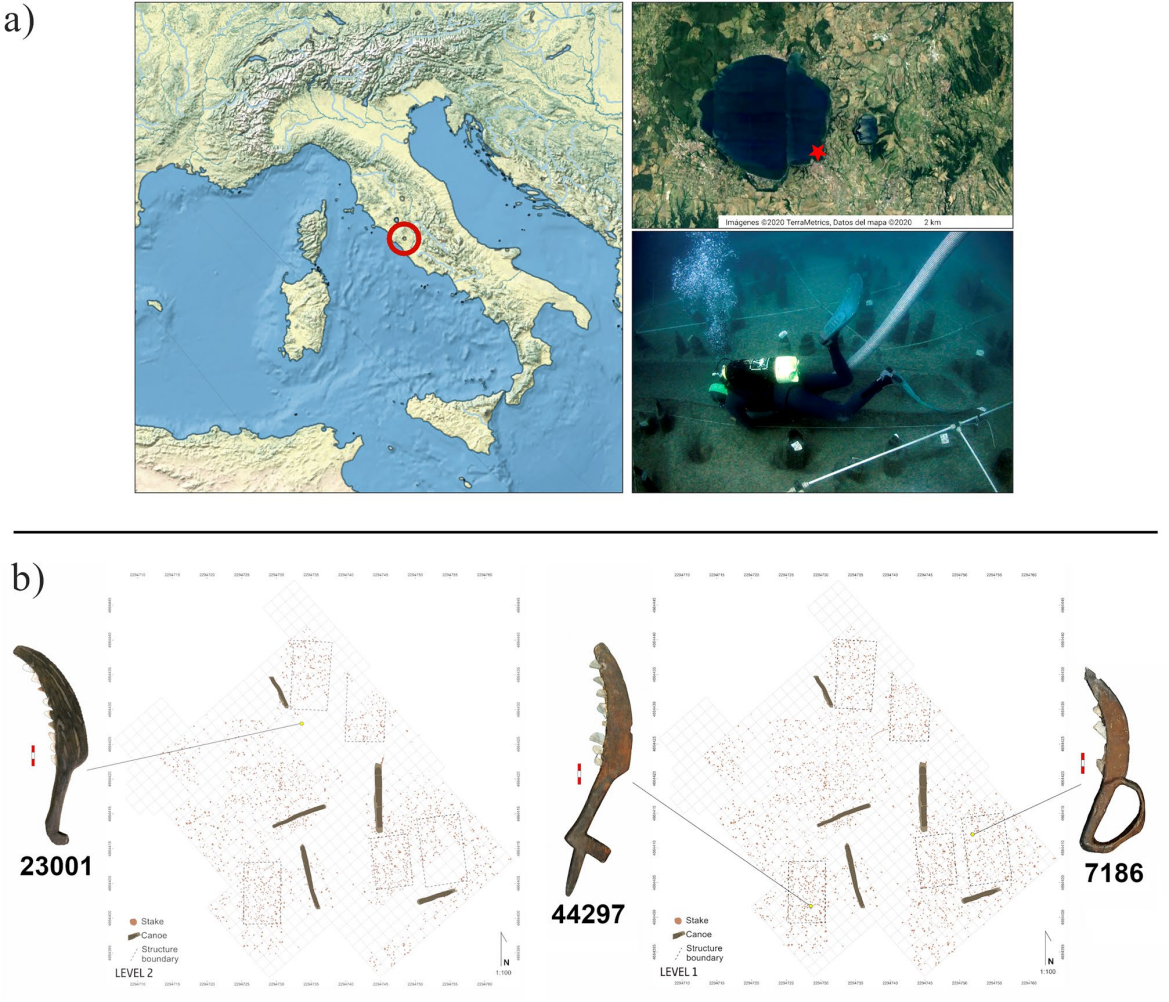
(**a**) Geographical position of La Marmotta. Photograph of the subaquatic excavation. Photo from Museo delle Civiltà (MuCiv); (**b**) position of the sickles within the two excavated layers (Layer I and II).

The early Neolithic site of La Marmotta is located under the waters of Lake Bracciano (Anguillara Sabazia, Rome, Italy). It has been excavated under the direction of M. A. Fugazzola Delpino (Soprintendenza Speciale, Museo Nazionale Preistorico Etnografico ‘L. Pigorini’, today Museo delle Civiltà)^26,27^. The archaeological deposit is located approximately 300 m from the modern shoreline, submerged at a depth of 11 m (8 m of water and 3 m of sediment), which has permitted exceptional conservation of the organic remains. The site covers an area of about 2 hectares, of which approximately 25% has been excavated (Fig. 1a). The stratigraphic sequence is composed of three contiguous layers (Layer II, Layer I, Layer ‘Chiocciolaio’), without any sterile layer or interruption between them^33^. Layer II corresponds to the beginning of the settlement. The pottery assemblage belongs to the Tyrrhenian facies of the Impressed Ware Culture. Layer I represents the time of the most intense occupation of the village. The pottery is here dominated by Cardial and Painted Ware. The last phase of Layer I, also called Layer ‘Chiocciolaio’, represents the abandonment of the settlement^26^. The radiocarbon dates from the wooden posts obtained so far are distributed over a time interval between 7840 and 7575 cal BP (post 213, square A2) and 7425–7010 cal BP (post 21, square B29)^34^. Similar dates have recently been obtained by dating two waterlogged poppy seed microsamples (7580–7420 and 7570–7420 cal BP)^35^. In the framework of the current research, six samples of charred caryopsides of emmer wheat (*Triticum dicoccum*) were dated (Table 1). Results confirm the data previously obtained; charred seeds from Layer II are dated between 7560 and 7330, while Layer I is dated between 7570 and 7165 cal BP. It might be speculated whether some of the dated samples (CNA-5289.1.2 and CNA-5291.1.1) were intrusive in their respective layers, but only with a larger series of dates will it be possible to define the chronology of the two main layers better.

**Table 1:** New radiocarbon dates on charred emmer wheat caryopsis from La Marmotta.

Lab. code	Sample n	Species	Layer	Square	Age (y)	± (years)	δ13C (‰)	pMC	Error	cal BP	
CNA-5754.1.2	24,837	*T. dicoccum* caryopsis	Liv. I Sup	A281	6367	34	− 23.92482	45.26737	0.18968	7420	7165
CNA-5288.1.1	19,985	*T. dicoccum* caryopsis	Liv. I Grigio	A25	6393	40	− 27.30331	45.11812	0.226555	7425	7175
CNA-5289.1.2	18,769	*T. dicoccum* caryopsis	Liv. I Grigio	A120	6577	43	− 21.39406	44.10007	0.236075	7570	7420
CNA-5290.1.1	28,564	*T. dicoccum* caryopsis	Liv. II	A274	6534	43	− 21.28299	44.33251	0.237435	7565	7330
CNA-5291.1.1	28,627	*T. dicoccum* caryopsis	Liv. II	A268	6431	42	− 21.84272	44.9088	0.23579	7425	7270
CNA-5755.1.1	26,126	*T. dicoccum* caryopsis	Liv. II	A235	6558	31	− 18.30000	44.20000	0.17000	7560	7420

#### The artefacts

**Figure 2:**
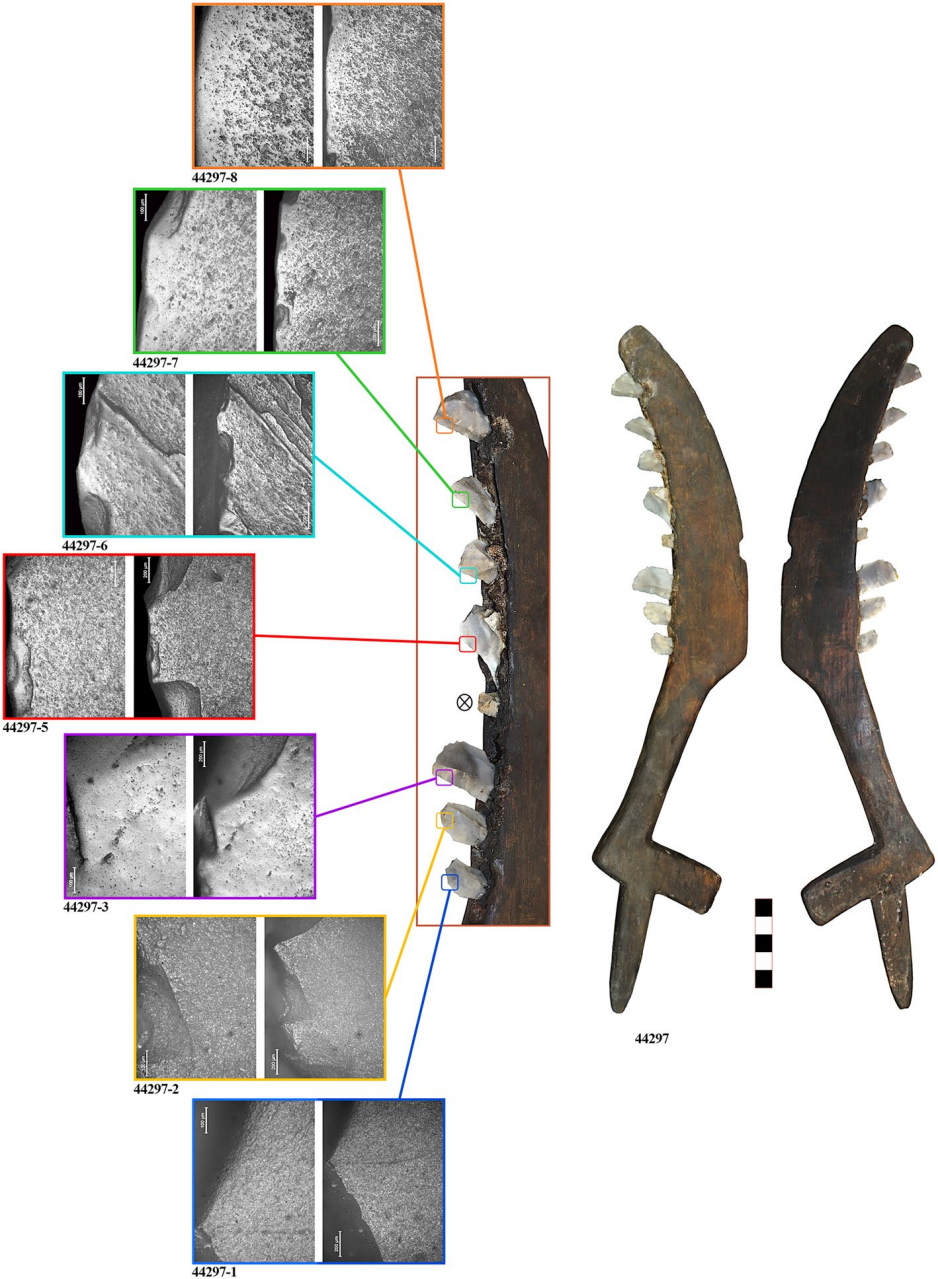
Sickle No. 44297 with macro and microscopic view of the lithic inserts (44297-1 to 44297-8). Micrographs have been taken at both 100 × and 200 × for each insert.

Sickle No. 23001 was found in the year 2000, in square A179 of Level II (Fig. 1b). The sickle is complete and still conserves nine stone inserts. The handle measures 8.5 (L) × 1.8 (W) × 1.2 cm (T) and is characterised by a hinged termination. The cutting edge measures 18.5 (L) × 4.5 (W) × 1.2 (T) cm, has a curved shape and on the inner side it was carved to create a space for placing the stone inserts, which measure between 0.9 and 1 cm in width. The tool was finely finished with smooth wooden surfaces and a decorative motif along the external ridge, characterised by a series of regularly distributed grooves (Fig. 2).

**Figure 3:**
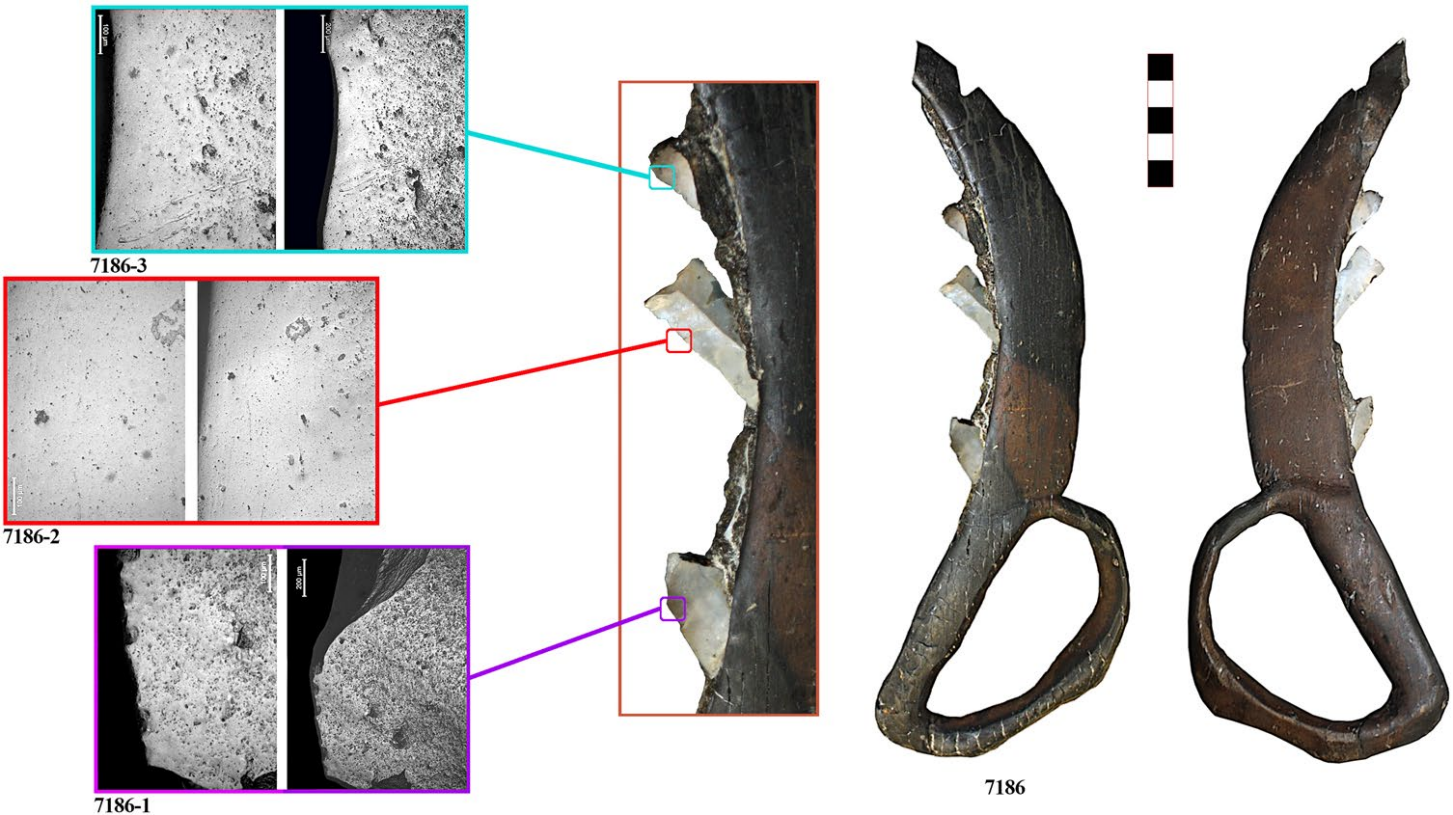
Sickle No. 7186 with macro and microscopic view of the lithic inserts (7186-1 to 7186-3). Micrographs have been taken at both 100 × and 200 × for each insert.

Sickle No. 44297 was found in 2005, in square A507 of Layer II (Fig. 1b). The complete sickle conserves eight stone inserts. The handle measures 18 (L) × 2 (W) × 1.6 cm (T) and is characterised by a ‘T’ shaped termination. The cutting edge measures 18.2 (L) × 4.2 (W) × 1.2 (T) cm, and has a curved shape. An incision between 1.0 and 0.7 cm in width was carved in it. It is characterised by smooth wooden surfaces (Fig. 3).

**Figure 4:**
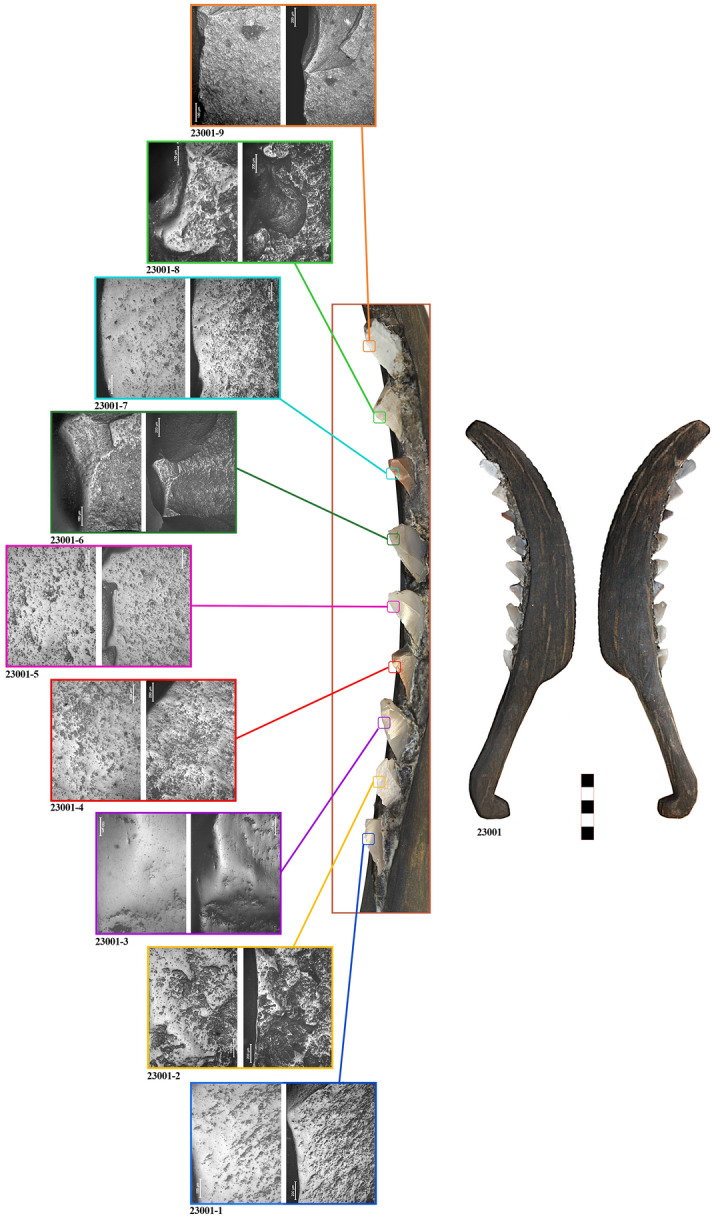
Sickle No. 23001 with macro and microscopic view of the lithic inserts (23001-1 to 23001-9). Micrographs have been taken at both 100 × and 200 × for each insert.

The third sickle, No. 7186, was found in 1996, in square D147 of Layer I (Fig. 1b). It conserves only three complete stone inserts. A fragment of the fourth insert is still visible inside the carved groove, but it is not possible to analyse it being too fragmentary. The handle measures 9 (L) × 5.9 (W) × 1.3 cm (T) and is characterised by an arched hand guard. The cutting edge measures 16.5 (L) × 3 (W) × 1.1 (T) cm, and has a markedly curved shape, with an incision of 1.0–1.3 cm width (Fig. 4).

All wooden artefacts were treated at the Laboratorio di Conservazione e Restauro at the Museo delle Civiltà (Rome, Italy). After excavation, for their conservation, artefacts were cleaned with demineralized water, and then treated with polyethylene glycol (PEG 400 and PM 4000) at different proportions (10%, 20%, 40%, and 80%) during a gradual restoration process that included cold and warm phases.

To obtain precise measurements and to avoid further manipulation of the artefacts, we built 3D digital models of the three sickles (Supplementary Information S1). We acquired sub-millimetric 3D geometric/colour data using a structured light scanner (model Breuckmann Smartscan-3D duo with colour cameras and sensors for inter-changeable 90, 150, 200 and 450 mm fields of view). The 3D capture equipment, in conjunction with modelling software (Rapidform/Geomagic and Meshlab), allowed the reconstruction of high fidelity (sub-millimetric) digital models of the sickles. Such models present a high level of geometric detail and they scale to the exact real size of the artefacts. We subsequently downgraded the raw, larger models (about one million polygons and half a million colour pixels on each mesh) to different levels of detail. These derivative models reflect different research aims (e. g., in most cases, models for geometric measurement do not require colour information; etc.). Models in 3D-PDF format for their visualisation and measurement have been included in supplementary information (Supplementary Information S8–S10).

### Methods

#### Experimental practice

In order to build an experimental interpretative framework, different harvesting experiments have been conducted. Three experimental sickles have been made; two of them with wood, *Quercus* sp., and one using antler. Experimental adhesive to fix stone inserts has been produced with a mix of pine resin (*Pinus sylvestris*), crushed pine charcoal, and beeswax. Stone inserts were made using different varieties of fine-grained tertiary flints. Three different experimental cereal fields were harvested: *Triticum aestivum* ‘Gentil rosso’ wheat^36^ in Tuscany (Italy) (15 h), *Hordeum vulgare* in Soria (Spain) (8 h), and *Triticum monococcum* in Provence-Alpes-Côte d'Azur (France) (10 h)^37^. Two experimental tools have been used for reaping common reeds (*Phragmites australis*) (2 h) and two tools to cut wild grasses (*Festuca sp.* and *Juncus sp.*) (1 h).

In addition, four other sickles were used to test low versus high height harvesting. Two sickles were used to harvest *Triticum dicoccum* in Asturias (Spain) (both for 6 h), and two others were used to harvest *Triticum spelta* also in Asturias (Spain) (one sickle used for 12 h and the other for 16 h). Therefore, the same cereal was harvested high with one sickle, near the spikelet, and low with the other, near the ground. More detailed information on the experimental practice is to be found in Supplementary Information S2.

#### Use-wear and microtexture analysis through confocal microscopy

Moulds of the lithic inserts fixed within the sickle handles have been made by using a two-component silicone paste (Provil Novo Light Fast by Heraeus Kulzer GmbH) that is commonly used for this goal in use-wear studies^38^. Prior to moulding, lithic surfaces were cleaned repeatedly with water, acetone, and alcohol and in order to remove superficial impurities, as well as to remove the polyethylene glycol used to restore the tools. Moulds were analysed at 100 × and 200 × magnifications using a Leica DM2500 M optical microscope for micro polish characterization with reflected light. Micrographs were taken with a Leica DFC420 C digital camera.

Experimental stone inserts were first cleaned with soapy water, using Derquim LM 01. Successively, inserts were immerged in a 10% H2O2 solution for 20 min’ bath in an ultrasonic tank to remove grease.

Polished areas from both archaeological and experimental inserts were scanned with the Sensofar Plu Neox blue light scanning confocal microscope, using a 20 × (0.45 NA) objective, with a spatial sampling of 0.83 μm, optical resolution of 0.31 μm, vertical resolution of 20 nm and a z-step interval of 1 μm. From 6 to 17 areas of 650 × 500 μm were scanned for each lithic insert. Afterwards, samples of 200 × 200 μm were selected from each area. Sampled areas were later processed with the SensoMAP Standard v.8 from Digital Surf in order to extract ISO 25178 texture parameters (Supplementary Information S11). Quadratic discriminant function analysis was used to treat extracted parameters, building a predictive model for group membership following a methodology previously described by Ibáñez and colleagues^39^. More detailed information on microtexture analysis is to be found in Supplementary Information S3.

#### Analysis of woody material: botanical identification

Taxonomic identification of the wood species was accomplished by observing the three anatomical planes of the wood (transversal, radial longitudinal and tangential longitudinal). The samples were obtained by extracting thin laminas from each of three planes of the wood with a cutting tool. The samples extracted must be of a size appropriate for the identification of species, but not too large as to damage the object^40–42^. The thin sections were observed with an optic microscope equipped with objectives with 4, 10, 20 and 50 × magnification (Leica DM 750 M at the Instituto Patagónico de Ciencias Sociales y Humanas: IPCSH-CONICET, Puerto Madryn, Argentina) and compared with a specialized atlas^43^. The treatment with polyethylene glycol has not impeded the taxonomic study a posteriori, as the microanatomy of the wood can still be observed, allowing taxonomic identification.

#### Organic residue extraction, GC and GC–MS analyses

Molecular analyses were conducted at the ORA Laboratory of the University of Tübingen (Germany) to investigate the organic residues which were used as hafting glue. The extraction and treatment of organic substances were carried out using the same method described elsewhere^43^ as well as gas chromatography (GC) and GC-Mass Spectrometry (GC–MS) analyses^45^. Briefly, ∼10 mg of residue was ground and solvent-extracted (Dichloromethane) for 30 min by ultrasonication to target lipid compounds. Each extract was trimethylsilylated by adding N,O-bis(trimethylsilyl)trifluoroacetamide (BSTFA, 50 μL) and a catalytic reagent (pyridin, 4 μL). Then, the trimethylsilylated samples were analysed by GC and GC–MS. More detailed information to be found in Supplementary Text S4.

#### Pollen and non-pollen palynomorph analysis

In total, seven samples of mastic were taken from the three sickles under investigation for palynological analyses. In order to preserve as much as possible the integrity of these unique and extraordinarily well-preserved artefacts, the amount sampled was very small, averaging 101 mg per sample (between 74 and 141 mg). The procedure to extract palynomorphs followed a protocol proven successful in several previous studies^46,47^. After dissolving in cold toluene for 24 h, the material was washed by centrifuging in the decreasing toluene-ethanol series and then in the increasing ethanol-deionised water series until hydration was complete. Standard acetolysis, carried out for 5 min at 100 °C^48^ and filtration through a 250 μm steel mesh were carried out. When a large amount of microcharcoal and mineral particles occurred, these were removed by “swirling” technique and dissolution with cold HF 40% for 24 h and hot HCl 10%^49^. The obtained residue was diluted to a known volume (0.5 mL) to enable calculation of absolute pollen frequency through sampling of 50–100 μL with a micropipette. Transmitted-light optical microscopy analysis was carried out at 400 × -600 × magnifications using a Leitz Diaplan, an Olympus BHS and a Vickers ML 1300 compound microscopes. Pollen and non-pollen palynomorph atlases and keys, reference collections and digital image archives were used for palynomorph determination^50–54^.

#### Phytolith analysis

Six samples were extracted from adhesive material for phytolith analyses, two samples for each sickle. Sickle 23001 was sampled from both an internal and external area, both near lithic 23001-6. Sickle 44297 was sampled in the mesial part, one external sample near lithic 44297-5, and one internal near lithic 44297-7. Sickle 7186 was sampled in the mesial part, both from the internal area near the broken lithic insert (Supplementary Materials S5). The methods used are similar to those described by Katz and colleagues^55^. A weighed aliquot of ca. 40 mg of air-dried and ashed sample (500 °C for 4 h) was treated with 50 µl of a volume solution of 6 N HCl and 450 µl 2.4 g/ml sodium polytungstate [Na6(H2W12O40)·H2O]. Microscope slides were mounted with 50 µl of material. Samples were examined at 200 × and 400 × magnification with an Olympus Bx43 optical microscope in the Archaeobotany Laboratory, Autonomous University of Barcelona. Morphological identification was based on standard literature^56–59^ and modern plant reference collections^60–64^. The terms used followed the International Code for Phytolith Nomenclature v. 2.0^65^.

## Results

### Use-wear and microtexture analysis through confocal microscopy

Microscopic observation of the silicone impressions through reflected-light microscopy identified well-preserved use-wear traces. The appearance of the traces closely matches the experimental traces obtained from plant harvesting (Figs. 2, 3, 4). In order to quantitatively determine whether sickle inserts had been used to cut domesticated cereals or other plants, glossy areas were studied by texture analysis of 3D images obtained through confocal microscopy. Five inserts were excluded from the analysis, two because they were made on coarse-grained flints (23001-2, and 23001-8), which could affect the classification, and three because they almost showed no use-wear traces (44297-1, 44297-2, and 44297-4). The results obtained for the remaining tools show that 14 of the 15 analysed inserts were classified as having harvested domesticated cereals (Supplementary Information S3), while only one insert was classified as having cut reeds. This latter insert (23001-9) shows very lightly developed wear and was probably added to the sickle in a later stage. As observed experimentally, the inserts placed at the distal end of the sickle are the ones most easily lost during harvesting as they are more exposed and, therefore, more likely to become detached after accidental impact with the ground, stones, etc.

The different degree of trace development reveals the existence of specific management strategies. While different degrees of trace developments can occur in the same sickle, depending on the position of each insert, in the case of sickle No. 44297 it is clear that the inserts were placed in the shaft at different times. The first two inserts (44297-1, 44297-2) were almost unused, while the third one shows a very well-developed gloss (44297-3). This suggests that the first two inserts had been replaced soon before the abandonment of the sickle, while the other inserts had been used for a considerably longer period. Similarly, the lithic 44297-4 is a residual insert from previous uses, and did not possess a functional purpose in the actual configuration of the sickle. Therefore, inserts were most likely not changed all at once, but at different times, possibly due to accidental loss of inserts during harvesting.

As can be seen from the micrographs, use-wear traces on the archaeological specimens display rather smooth polish, with a slightly pitted appearance and thin striations. This is clear especially in the case of inserts with well-developed gloss (23001-1, 23001-5, 7186-2, 7186-3, and 44297-3). The presence of striations and abrasions is usually associated with low height harvesting, because of the occasional contact of the lithic inserts with ground particles^66^. This has been confirmed by the quantitative analysis of experimental and archaeological use-wear traces, as 93.3% of the analysed sub-areas on the La Marmotta sickles are classed together with experimental tools used for low height harvesting, at the base of the stem^39^ (Supplementary Information S3).

### Analysis of woody material: botanical identification

**Figure 5:**
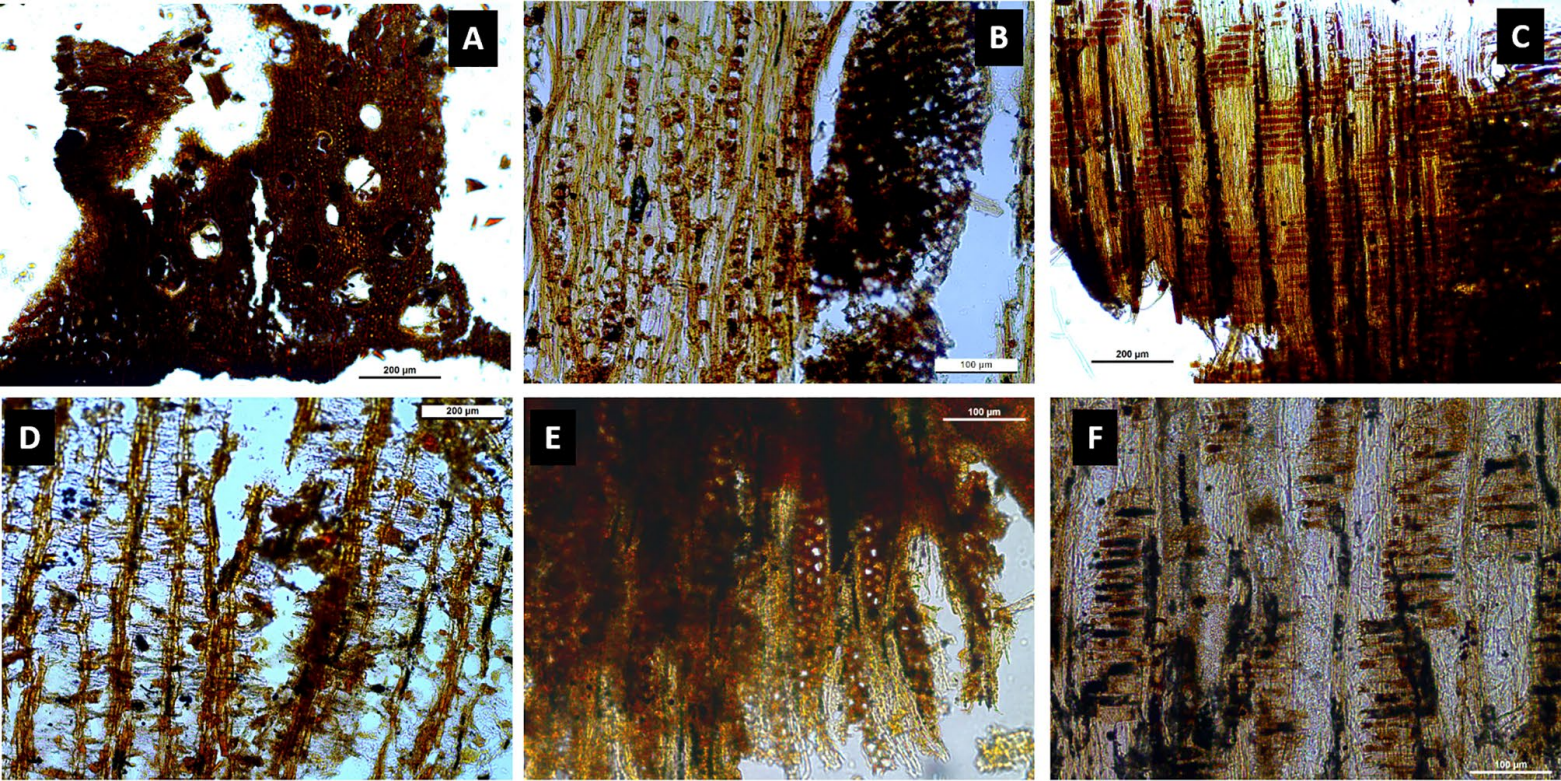
(**A**) Sample from the transversal section of deciduous *Quercus* sp.; (**B**) sample from the tangential section of deciduous *Quercus* sp.; (**C**) sample from the radial section of deciduous *Quercus* sp.; (**D**) sample from the transversal section of *Rosaceae*/*Maloideae*; (**E**) sample from the tangential section of *Rosaceae*/*Maloideae*; (**F**) sample from the radial section of *Rosaceae*/*Maloideae*.

The taxonomical analysis of the three sickles evidenced the use of two woody species as raw material (Fig. 5). Two of them were made with wood from deciduous oak (*Quercus* sp.) (No. 44297 and No. 23001) and one with wood from Rosaceae/Maloideae (No. 7186). It should be noted that the taxonomic analysis of other types of artefacts at La Marmotta, also has equally shown the use of deciduous *Quercus* sp. in their fabrication (publication in prep.). However, the use of Rosaceae/Maloideae had not been identified at the site for other types of artefacts, connected with sailing or hunting, for instance. Despite that, the use of deciduous oak wood is well-documented at the site overall^67,68^. Although Rosaceae/Maloideae has not been identified as a raw material for other types of artefacts, this taxon had been documented at La Marmotta. According to Fugazzola’s studies, the inhabitants of the village knew several fruit tree species, including crab apples^26,67^.

### GC–MS analysis

The identification of characteristic diterpenes markers from the abietane and pimarane families in the adhesive samples indicates the use of Pinaceae resin(s)^69–71^ to haft the three sickles from La Marmotta. Dehydroabietic, abietic, isopimaric and sandaracopimaric acids were detected in all of them (Supplementary Information S4). In addition, the presence of Seco- dehydroabietic (α and β) and pimaric acid as main pimarate suggest a pine origin^69^ for the resin on sickle 23001.

### Pollen and non-pollen palynomorph analysis

**Figure 6:**
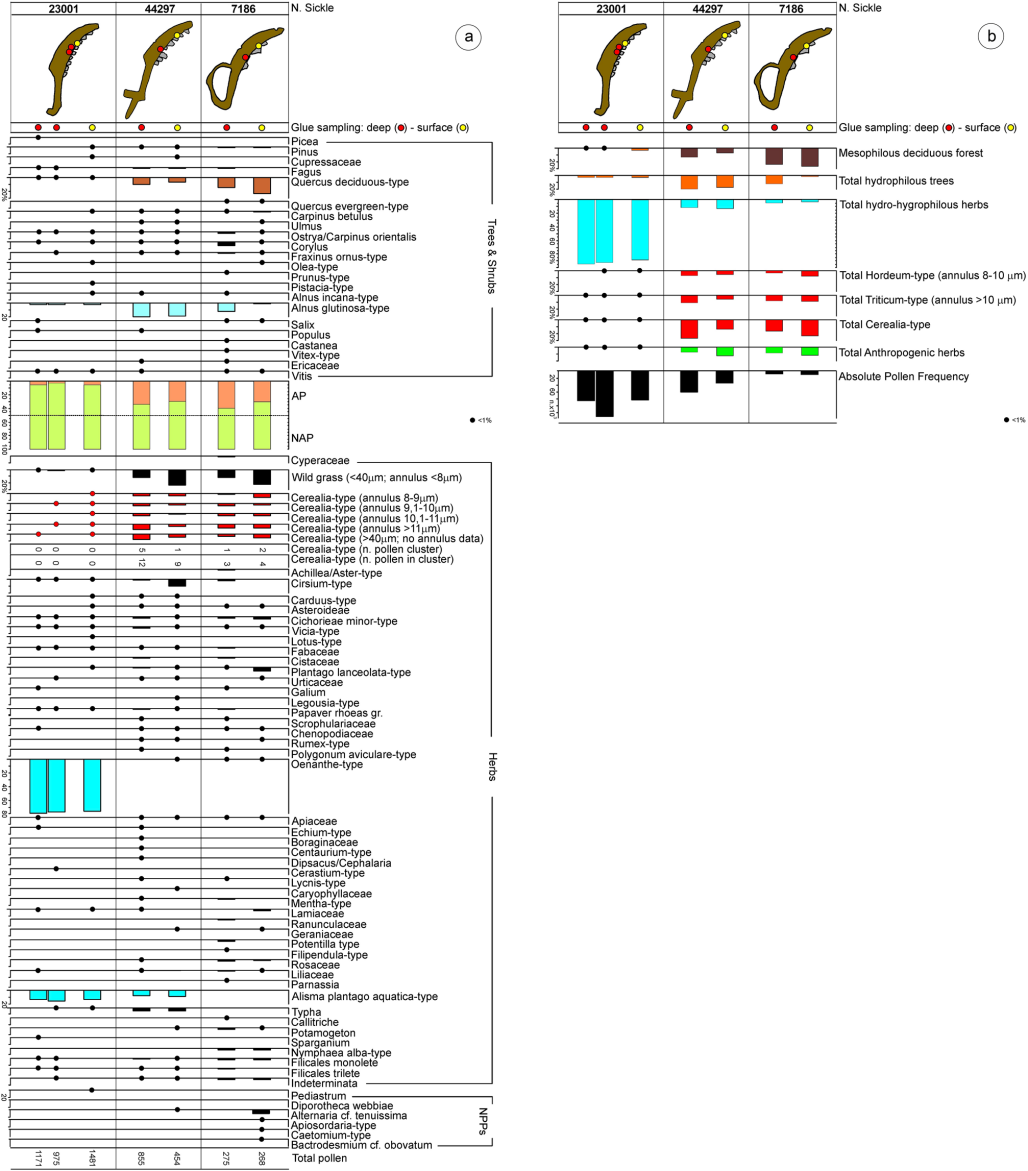
(**a**) Pollen percentage diagram of Arboreal Pollen (AP), Non-Arboreal Pollen (NAP) taxa, and non-pollen palynomorphs (NPPs) in the adhesive on three sickles from La Marmotta. NPP values are expressed as percentages of total pollen; (**b**) pollen percentage diagram of selected categories of ecological and anthropogenic interest in the adhesive on three sickles from La Marmotta.

Mastic was sampled both from the outer surface, in the gaps between lithics, and at the depth of a few millimetres, inside the hafting grooves, in order to assess potential qualitative and quantitative differences in the palynological content at different depths. The pollen absolute frequency was variable (between 6501 and 133,929 grains/g), and usually higher in the inner samples compared with the outer ones. Each sample, mostly containing a large amount of well-preserved pollen, has allowed counting, on average, more than 780 grains (from 271 to 1481). Figure 6 show percentage values of individual arboreal and shrubby taxa (AP), herbaceous taxa (NAP) and NPPs, as well as cumulative data for plant categories, which are relevant in terms of ecology and anthropogenic impact.

The first two sickles (No. 7186 and 44297) suggest a very similar palynological scenario. Among the arboreal taxa (AP between 29.5 and 39.1%), oak (*Quercus* t. *caducifolia*) (6.4–23.5%) prevails, with a sporadic occurrence of other elements in the mesophilous deciduous oakwood, including *Carpinus betulus*, *Ulmus*, *Ostrya/Carpinus orientalis*, *Corylus*, *Fraxinus ornus*-type and *Vitis*. Typically, Mediterranean evergreen species, such as *Quercus ilex*-type, *Olea*-type and *Pistacia*, play only a minor role in this assemblage.

Among the hydrophilous arboreal species *Alnus glutinosa*-type deserves a mention, followed by *Salix* and *Populus*. More widely documented are grains of a number of taxa probably common in the riparian and aquatic habitats of the site, such as *Parnassia*, *Alisma plantago aquatica*-type, *Typha*, *Callitriche*, *Potamogeton*, *Sparganium* and *Nymphaea alba*-type. *Potamogeton*, *Sparganium* and *Nymphaea* have been documented also from the analysis of macroremains (Supplementary Information S7).

Wild grasses occur in particularly high percentages, which reach identical values in the adhesive from the two sickles, in both cases with higher values in the superficial levels of the mastic (between 22.0 and 23.1%) than in deep samples (between 11.4 and 11.6%). Analogous considerations apply to the herbaceous anthropogenic taxa typical of cultivated and ruderal areas (*Cirsium*-type, Cichorieae *minor*-type, *Plantago lanceolata*-type, Urticaceae, *Galium*, *Papaver rhoeas* gr., Chenopodiaceae, *Rumex*-type. and *Polygonum aviculare*-type)^72,73^.

In order to separate different cereals, the biometric data of each individual pollen grain have been evaluated, measuring the average diameter and the pore annulus. The examination, in spite of some limitations that cannot always avoid equivocal determinations^51,52,74–77^, have allowed identification of both *Triticum*-type (d > 50 μm and annulus > 10 μm) and *Hordeum*-type (d = 40–50 μm and annulus 8–10 μm), occurring with values of 5.5–10.6% and 4.4–8.6%, respectively. Generally, percentages of Cerealia are highly relevant in all samples (between 15.4 and 26.0%). Both cereals often occurred also as pollen aggregates.

The results obtained from the three mastic samples of sickle No. 23001, one taken from the surface and two from within the adhesive, appear entirely different and atypical compared with the two previous artefacts. Arboreal taxa occur in minimal amounts (AP between 2.7 and 4.7%), among which *Alnus glutinosa*-type prevails (values between 1.6 and 2.4%), along with equally low percentages of Cerealia (between 0.2 and 0.9%) and anthropogenic indicators (averaging 0.8%), whereas grains of swampy herbaceous plants occur in constantly high percentages, notably *Oenanthe*-type (averaging 78.1%), and to a lesser extent *Alisma plantago-aquatica*-type (averaging 13.8%).

Based on preliminary observations made exclusively using optical microscopy and concerning the main morphological and biometric characteristics of 50 grains of *Oenanthe*-type [P = 33.8-(39.4)-48.4 μm; E = 14.2-(19.4)-22.4 μm; P/E = 1.7-(2.0)-2.5 μm; PAI = 0.22-(0.30)-0.39 μm], and according to the data reported in lietrature^78^, their attribution to *Oenanthe aquatica* group and to *Oenanthe peucedanifolia* group should be considered valid.

Most of samples were devoid of NPPs, with the exception of No. 23001 and 7186 (the samples from the external surfaces). No. 23001 included algal remains (*Pediastrum*), whereas No. 7186 contained five fungal taxa (*Alternaria*, *Apiosordaria-*type, *Chaetomium-*type, *Bactrodesmium*, *Diporotheca webbiae*), the first of which is by far dominant. Due to their size (c. 50–55 μm), the typically club-shaped spores of *Alternaria *can be identified as conidia of *A. tenuissima*^79^, while the morphology of *Bactrodesmium* spores points to conidia of *B. obovatum* or *B. abruptum*^80^.

### Phytolith analysis

**Figure 7:**
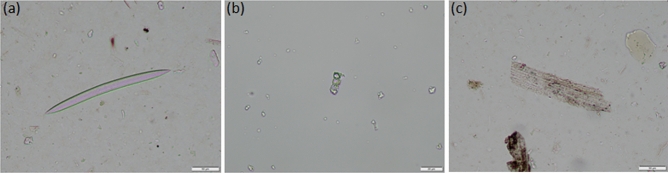
Photomicrographs of phytoliths and other siliceous microfossils identified in the samples (200 × or 400 ×). (**a**) Sponge spicule; (**b**) articulated spheroid phytoliths from dicotyledonous plants; (c) multicelled elongate phytoliths from grasses.

Phytoliths were recorded in all the adhesive samples in different amounts (ranging from 84,000 to 470,000 phytoliths per 1 g of material Supplementary Table S5). In addition, other siliceous microfossils such as diatoms and sponge spicules were also noted in the phytolith slides (Fig. 7). Their presence in association with phytolith abundance points towards general good preservation conditions of silica assemblages.

Most of the phytoliths from inner samples derived from dicotyledonous plants, mainly from wood/bark with an average of about 60% (Supplementary Table S5). The dominant morphotypes were spheroids, displayed either singly and in articulated groups (Fig. 7). Spheroid phytoliths occur in a range of plants including woody dicotyledonous (in the sense of non-monocot angiosperms) and some herbaceous monocots^64^, but in particular, ornate surface textures (rugose or scabrate) are produced in variable amounts by woody taxa from Mediterranean areas, such as (*Pinus* sp.) and oak (*Quercus* sp.)^59,64^. The phytolith records are consistent with biomarker signatures pointing to a pine origin of the adhesive, and therefore also with the sickle wood raw material (deciduous *Quercus* sp. in samples 44,297 and 23,001).

Of particular note is the presence in all the samples of phytoliths from the culms and leaves of Pooideae grasses (Fig. 7c), which are common in wetlands and include cereals such as wheat and barley reported in the site’s macrobotanical records, including hulled barley (*Hordeum distichum*), emmer (*Triticum dicoccum*), einkorn (*Triticum monococcum*) and free-threshing wheat (*Triticum turgidum* or *Triticum durum*)^81^.

## Discussion

This study presents new data for three of the most complete sickles in the Early Mediterranean Neolithic, dated between *ca.* 7560 and 7165 cal BP. Combining use-wear, archaeobotanical, and residue analysis it has been possible to gain insight into the earliest evidence for harvesting practises in the Central Mediterranean Neolithic, but also into the environment around the site and the cereals cultivated.

The three sickles are rather small, between *ca.* 36 and 24 cm of maximum length, with a cutting edge between 18 and 16 cm long. This size is similar to the so-called Karanovo antler sickles from Bulgaria, dated between *ca.* 8200–7500 cal BP, whose cutting edge is between 24 and 14 cm long^82^. Identical specimens have been recovered at the sites of Kovačevo^83^, also in Bulgaria, and Hacılar VI^84^ in Turkey. In comparison with historical and ethnographic iron sickles, it seems that Neolithic harvesting tools were smaller and adapted to cutting a small number of plants with each stroke^85,86^. The length of the blade is, indeed, directly related to the quantity of plants to be harvested^29^. For example, traditional sickles employed in Eurasia during the eighteenth–nineteenth centuries generally had a blade of about 30–40 cm or more^87^.

The ligneous raw material used for their manufacture (deciduous *Quercus* sp. and Rosaceae/Maloideae) were not exclusively employed for sickles. Both species were widely used at La Marmotta for a diversity of wood crafts. On one hand, the frequent use of deciduous *Quercus* sp. wood at the site may be related to the frequency of the taxon in the local vegetation during the Neolithic. Pollen records from the nearby Lago di Vico and Lagaccione (Lago di Bolsena), further to the north, show that abundance of deciduous *Quercus* sp. was a key feature of the mid-Holocene vegetation^88^. On the other hand, the wood of Maloideae would have been relatively easy to acquire during forest clearings. The wood of the different species of the Maloideae is hard and characterised by close, compact grain that makes it ideal for carving and engraving. At other prehistoric sites, the wood of Maloideae has been used for artefacts such as axe hafts, spear shafts and tool handles^89–91^, while at La Draga site, in north-eastern Iberia, the use of Rosaceae/Maloideae as a raw material has only been documented in the remains of charcoal associated with the use of firewood^92^.

All sickles show a very specific design and particular attention was paid to their shaping and finishing. As experimentally tested, the arched hand guard of sickle No. 7186 enables a tighter control over the tool during harvesting. Still puzzling is the ‘T’ shape termination of sickle No. 44297 and more detailed experimental and use-wear studies are necessary. In addition, it would also be important to determine whether the heterogeneity in the use of ligneous raw materials reflects differences in the type of haft (i.e., hinged handles, handles with hand guard, etc.). Ongoing archaeobotanical studies, based on techno-morphometric analyses, will be able to assess whether the taxonomic selection was connected to the morphology of the different sickles.

Stone inserts are mainly made on blade fragments, as visible from Figs. 2, 3 and 4. Their typological and morphometric characteristics are like the rest of the sickle inserts found in the flaked stone assemblage of La Marmotta (Supplementary Information S6). Truncations are dominant, average sizes are 29–20 mm in length, 13–10 mm in width, 3–2 mm in thickness. Based on a macroscopic observation, a provenance from Gargano promontory can be suggested for some of the stone inserts included in the wooden sickles (e.g. 44297-5). However, regional varieties of Apenninian chert types are the most used raw-materials^93,94^. The flaked stone assemblage recovered at La Marmotta is made on these same raw materials, indicating that the site was integrated in regional and supraregional networks of chert circulation. Obsidian provenance^95^ data confirm that the site was embedded in a far-reaching network of raw-material distribution.

The use of Pinaceae resin for hafting is confirmed both by GC–MS analysis and by the two microfossil records (pollen and phytoliths). Pinaceae resin is not common in archaeological contexts, and it probably represents the first evidence of its use as adhesive substance during the Neolithic. The only other known case of the use of Pinaceae resin for hafting is from two Palaeolithic cave sites in South Italy^96^. Mediterranean pine was available in the site surroundings, as well as documented by pollen analysis from neighbouring lakes^88^, pointing to a local or regional procurement. This local supply strategy for adhesive materials seems to confirm practices previously documented in Central Italy in the eighth millennium cal BP. Indeed, local bitumen was used for the same function in the region between the Adriatic Sea and the Apennine Mountains^97,98^. Nevertheless, and although rarely documented, the use of exogenous materials to produce adhesives has recently been identified for the Mediterranean Neolithic. This is the case of birch bark, whose use is documented over a large area from Catalonia to the Apennines^43,98,99^.

The analysis of Neolithic mastic from the site of La Marmotta has shown the capability of this material to trap a remarkable amount of pollen grains, which appeared to be in an excellent state of preservation^46^. Therefore, this material has the potential to provide new research insights and data on the vegetation scenario around a site, the cultivated taxa and the local synanthropic flora.

The results obtained from the first samples (No. 7186 and 44297), rich in individual or grouped *Triticum*-type and *Hordeum*-type grains, further confirm the reaping function of these tools. Indeed, it is widely known that cereal pollen has a tendency to stick to the caryopses, in particular underneath the glumes of hulled wheat until spike maturation occurs, and that these grains can be found in high numbers within the harvest and even in the by-products of cereal grinding^75,100–103^. In contrast, the exceptionally high concentration of *Oenanthe*-type pollen in the third sickle (No. 23001) is unusual and unexpected. The genus *Oenanthe* includes hydro-hygrophilous plants in wet meadows, swamps and lakeshores, and it is still present in the Tyrrhenian area of central Italy. Due to the content of oenanthotoxins in the fruits and roots, several species of *Oenanthe* genus possess pharmacological properties. These substances have active principles similar to the cicutoxin of *Cicuta virosa*, with a neuro-enterotoxic effect. Some species, when consumed fresh and even in small quantities, seem to be able to induce a mental state similar to drunkenness, but can also cause nausea, vomiting and convulsions; but they are harmless when consumed dry^104–106^. *Oenanthe* plants have long been used as medicinal plants in Asia^107^. In an eighteenth century herbarium, however, it is named as *Cicuta palustre*. The caption near the specimen explains that, due to its toxicity, it is “*…stirps ipsa venenata infamis”* (known for its poisonous properties)^108^.

This anomalous find lacks an easy interpretation at the moment and can be added to the list of ‘unusual’ taxa identified at La Marmotta, so far known through the macrofossil record, such as *Papaver somniferum*, *Carthamus lanatus* and *Silybum marianum*, which are species with officinal and medical properties. In this sense, in agreement with Rottoli^109,110^, it cannot be ruled out that the first Neolithic communities settled on the shores of Lake Bracciano managed floral resources in various ways, and not only for strictly nutritional purposes. Aside from cereal crops (*Hordeum disticum*, *Triticum dicoccum*, *Triticum monococcum*, *Triticum durum/turgidum*) and legumes (*Pisum sativum*, *Lens culinaris*, *Lathyrus cicera/sativus*, *Vicia cf. sativa*, *Vicia* cf*. ervilia*, *Vicia* cf*. faba*) documented in the macrobotanical records, a number of officinal plants may have been harvested around the lakeshore village of La Marmotta for medicinal, tanning and textile purposes, and also for various craft works, the exact ethnobotanical significance of which it is difficult to unveil. The potential psychoactive effects linked to the “drunkenness” effect provoked by the active principles of *Oenanthe* may have been part of these medical or ritual uses.

Pollen results for sickles No. 7186 and 44297 are in agreement with data provided by the quantitative analysis use-wear traces. Use-wear traces are grouped with experiments harvesting domesticated cereals (*Hordeum vulgare*, *Triticum monococcum* and *Triticum aestivum*). Nevertheless, the high percentage of *Oenanthe*-type pollen on sickle No. 23001 is not fully reflected by the use-wear traces on the inserts of this same tool, which is grouped with the experimental harvesting of domesticated cereals. This discordancy between pollen and use-wear may be a consequence of two different factors:

- it is possible that the accumulation of *Oenanthe* pollen is the result of a last, single harvesting, while the use-wear pattern represents the accumulation of several previous, repeated, harvesting episodes. The presence of a minor component of Cerealia-type pollen in the adhesive from No. 23001 (Fig. 6) and of phytolith morphotypes derived from the leaves and stems of grasses of the Pooids (Supplementary Information S5) are consistent with the hypothesis that sickle No. 23001 was used for harvesting cereals at some point. In addition, it is worth remembering that insert 23001-9, likely the last one to be added to the sickle given the low degree of use-wear development, is classed together with experimental tools used for cutting reeds, plants growing in moist, swampy areas, as also many plants of the *Oenanthe* genus. Therefore, the use-wear traces on 23001-9 may represent the outcome of a last harvesting episode in which the sickle was used for cutting non-cereal plants, such as plants of the *Oenanthe* genus, but other also plants in moist, swampy areas. Among the macrobotanical record, for example, fragments of the rhizome of *Phragmite australis* are abundant (Supplementary Information S7). It is also remarkable that sickle No. 23001 is the only one among all the La Marmotta wooden sickles with a decoration on its back, and a special function for this tool may be imagined.
- another possibility is that the presence of pollen from aquatic plants is due to the depositional conditions from which tools have been recovered. The presence of moist habitats is well indicated by the abundance of aquatic pollen grains and is paralleled by the occurrence of *Pediastrum*, a green alga colonising eutrophic water pools^111^, of *Diporotheca webbiae*, a fungus parasitic on marsh ferns^112^, and of *Bactrodesmium*, a fungal genus thriving especially on submerged wood. In addition, the presence of diatoms and sponges also confirms that siliceous remains occurring in freshwater habitats were embedded within the resin.

Plant micro remains (phytoliths, pollen, NPPs) trapped into the adhesive also contribute to a reconstruction of the environment near the harvested fields. The palynological record indicates the presence of a Mediterranean deciduous forest in the surroundings, but also with species typical of aquatic habitats. The presence among NPPs of *Bactrodesmium obovatum* and *B. abruptum*, both saprophytic on arboreal taxa such as *Quercus*, *Fraxinus*, *Acer*, *Fagus*, *Alnus*, *Betula*, *Populus*^79^, also points to forested areas in the immediate vicinity of the field, as fungal spores have a limited spatial dispersal compared to pollen grains. Interesting is the presence of spores of *Apiosordaria*-type and *Chaetomium*-type, which although occurring in very low amounts, are often associated with herbivore dung and herding activity^113^. The faunal record from La Marmotta is based on domesticates, mainly goat and sheep, along with cattle, pigs and dogs, as well as wild animals including red deer, red fox, birds, reptiles and fish^26–28^.

Wild grasses are incorporated in the adhesive as well, particularly in sickles No. 7186 and No. 44297; in both cases with higher values in the superficial levels of the mastic (between 22.0 and 23.1%) than in deep samples (between 11.4 and 11.6%). Analogous considerations apply to the herbaceous anthropogenic taxa typical of cultivated and ruderal areas (*Cirsium-*type, *Cichorieae minor*-type, *Plantago lanceolata*-type, Urticaceae, *Galium*, *Papaver rhoeas* gr., Chenopodiaceae, *Rumex*-type and *Polygonum aviculare*-type)^72,73^. These data suggest that the harvested cereal fields were characterized by a greater diversity of weeds than inferred from the on-site macrobotanical record at La Marmotta (Supplementary Information S7). Harvested crops probably underwent several cleaning stages before being stored at the site.

The low height harvesting documented through the qualitative and quantitative study of use-wear traces confirms that the entire plant was cut and exploited, not only the ears. Cereal straw was probably part of the materials used for building housing structures and different crafts at La Marmotta. Nevertheless, despite the abundant and exceptionally preserved archaeobotanical record at the site, a full and detailed study of basketry, textiles, ropes and other aggregates of vegetal fibres is still to be addressed. Preliminary analysis of the charred seed assemblage indicates that the percentage of culms within the carpological assemblage is very low. However, as mentioned above, this might be due to crop cleaning activities carried out outside the village.

Through the analysis of microremains trapped in the adhesive it has been possible to obtain a better picture of the floral scenario around the dwelling area and the crop fields, to date investigated through more common approaches (e.g., palynological and macrobotanical records from archaeological sediments). Nevertheless, some caution is necessary when interpreting results, as adhesives have undergone a series of manipulations over time: from their hot preparation, obtained mixing several ingredients of different origin (pine resin, charcoal powder, etc.), to their use for hafting lithics inside the groove cut into the sickle and their exposure to the external environment during agricultural activities, and after that, to depositional and post-depositional agents. Moreover, these tools may have had a long lifetime, perhaps extending over a few decades, and they may have been periodically fixed, due to inevitable wearing-out of lithic blades and consequent replacement, to maintain efficiency of use. Evidence for this has also been provided by experimental studies (Supplementary Information S2), which have shown that these sickles need frequent mastic replacement during the use, in order to keep the blades in place and prevent them from detaching and moving. This action can easily transport organic material towards the inner layers of the mastic, which is eventually trapped in the longitudinal groove of the tool. Therefore, also based on experimental research, it is reasonable to hypothesize that most of the residues observed in the adhesive were incorporated during harvesting activities. The sickles trapped inside and on their surfaces a wide range of microparticles of botanical origin, including pollen grains, fungal spores, microalgae, fragment of leaf epidermis and other tissues, as well as microorganisms and dust particles.

## Conclusions

The wooden sickles from the submerged site of La Marmotta are some of the most impressive archaeological finds associated with the expansion of the Neolithic into the Mediterranean. This contribution provides significant insight into the life of these tools, from their production to their use.

The multiproxy approach carried out integrates, for the first-time, use-wear and microtexture analysis of stone tools, with taxonomical analysis of wood, GC–MS analysis of the adhesive substances, along with direct microfossil evidence from pollen, NPP and phytoliths. It has considerably improved our knowledge of Early Neolithic harvesting and farming systems. Sickles are emblematic tools whose manufacture and use are embedded in a larger economic system including the procurement and crafting of exogenous and local raw materials, plant food processing and production, as well as possibly medicinal and psychoactive behaviours.

## Supplementary Information

Supplementary Information 1.

Supplementary Information 2.

Supplementary Information 3.

Supplementary Information 4.

Supplementary Information 5.

Supplementary Information 6.

Supplementary Information 7.

Supplementary Information 8.

Supplementary Information 9.

Supplementary Information 10.

Supplementary Information 11.

## Data Availability

All data generated or analysed during this study are included in this article as supplementary information files.
