# Supplementary material for: Multiproxy study of 7500-year-old wooden sickles from the Lakeshore Village of La Marmotta, Italy
Source: Sci Rep. 2022 Sep 2;12:14976. doi: 10.1038/s41598-022-18597-8 (PMC9440057; doi:10.1038/s41598-022-18597-8)
Supplement: Supplementary file 1 — Supplementary Information 1. [file 41598_2022_18597_MOESM1_ESM.docx]

# S1. 3D Scanner Models.

All the three sickles presented in this work, were scanned using a second-generation Breuckmann SmartScan3D structured light scanner, with interchangeable 150 and 90 mm objectives. These are the type of objectives recommended for small and medium-sized objects when using this equipment. They allow obtaining dense clouds of high precision points (sub-millimetre error, usually less than 0.2 mm). At the end of the process, it allowed us to reconstruct the 3D surface of the objects with great precision and always with the exact information of the real measurements.

After the scans, we reconstructed the 3D models successively using Breuckmann's proprietary software (data export), the Rapidform/Geomagic (trademarked) and Meshlab (Free, Open Source) programs to finish the models.

In this supplementary information, we included the 3D models in PDF format, with sparse point clouds, to make them manageable in size. We generated the 3D-PDF with Adobe Acrobat X Pro.
