# Supplementary material for: Multiproxy study of 7500-year-old wooden sickles from the Lakeshore Village of La Marmotta, Italy
Source: Sci Rep. 2022 Sep 2;12:14976. doi: 10.1038/s41598-022-18597-8 (PMC9440057; doi:10.1038/s41598-022-18597-8)
Supplement: Supplementary file 2 — Supplementary Information 2. [file 41598_2022_18597_MOESM2_ESM.docx]

# S2. Experimental practice.

*Harvesting Triticum aestivum.* An old variety of *T. aestivum*, cv. ‘Gentil rosso’ wheat, has been harvested in 2019 in Tuscany, in a field near the village of Montespertoli. Field was cultivated by a local association, ‘Grani Antichi di Mosterpertoli’, focused on the recovery of an ancient wheat species, the ‘Gentil rosso’. Gentil rosso is a bread wheat, native to Central and Northern Italy, and spread in the mid-1800s in the region of Tuscany and Emilia due to its high productivity. Grain has been harvested with an experimental sickle realized on *Quercus* sp. taking sickle MA-187789 as a reference model. Originally, sickle had nine lithic inserts, but one of them was lost during the harvesting session. Lithic inserts have been shaped by abrupt direct retouch from bladelets of different varieties of fine-grained flints (i.e. Gargano and Tuscan Apennines – Italy; Fécamp – France) and one obsidian flake from Caucasus. Approximately 400 m2 were harvested for 3 days (15 hours of work), for a total of 150 kg of wheat. Grain has been harvested with a low height cutting, though without any direct contact between the sickle and with the soil. When harvested, grain was in a hard dough stage of ripening.

**
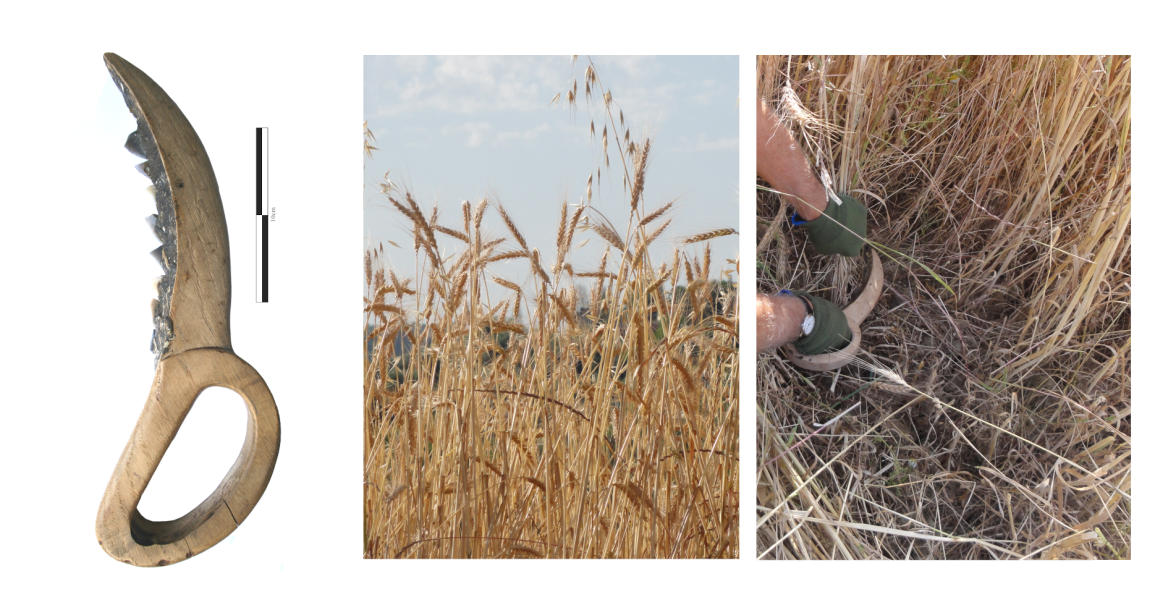
**

Figure S2-1. Experimental sickle and harvested field of *Triticum* *aestivum* cv. ‘Gentil Rosso’ (Italy)

*Harvesting Triticum monococcum.* Einkorn wheat was harvested in August 2013, 2015 and 2016 near Séranon in the Provence-Alpes-Côte d'Azur, in France. Grain has been harvested in a ripe state, with a curved sickle. Three unretouched fine-grained flint blades were inserted in a parallel way in an antler haft and fixed with bitumen. Approximately 80 m2, corresponding to 40.000 stems, have been cut for 10 hours. Grain has been harvested with a low height cutting, though without any direct contact between the sickle and with the soil.


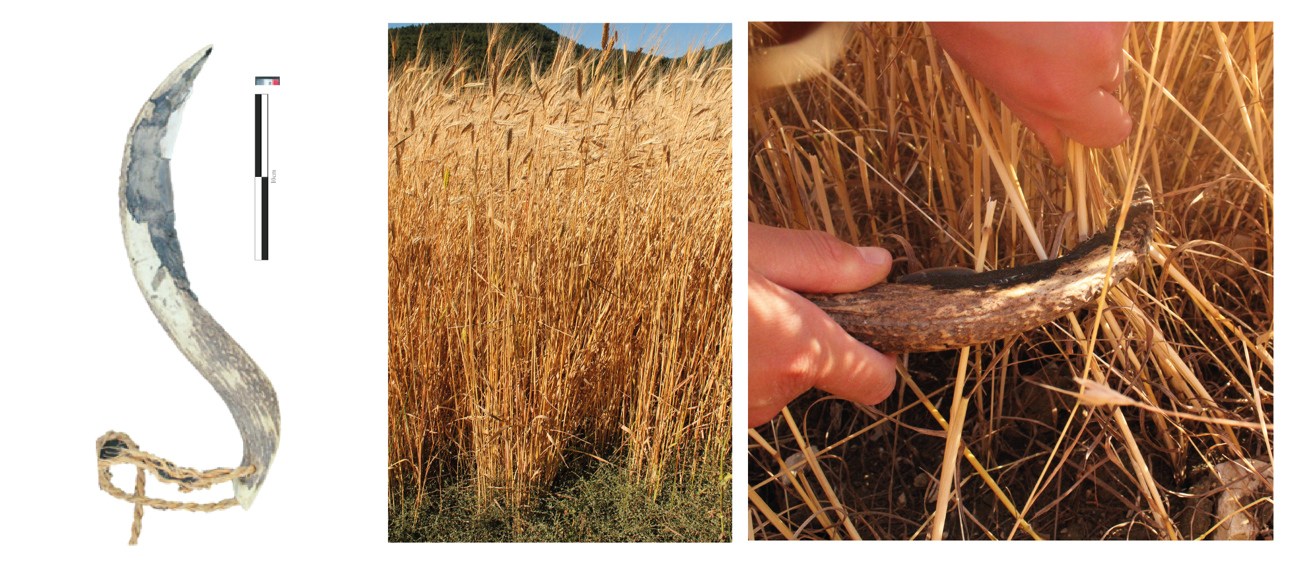


Figure S2-2. Experimental sickle and harvested field of *Triticum* *monococcum* (France)

*Harvesting Hordeum vulgare.* Barley was harvested in July 2020 near the village of Reinoso, Burgos (Spain). Grain has been harvested, with an experimental sickle realized on *Quercus* sp. taking sickle MA-187882 as a reference model. Sickle had seven inserts. Lithic inserts have been shaped by abrupt direct retouch from bladelets of different varieties of fine-grained flints (i.e. Gargano and Tuscan Apennines – Italy; Fécamp – France) and one obsidian flake from Caucasus. Approximately 300 m2 were harvested for 2 days (8 hours of work). Grain has been harvested in a fully ripe stage, through a low height cutting. However, the stone inserts were not directly in contact with the soil.


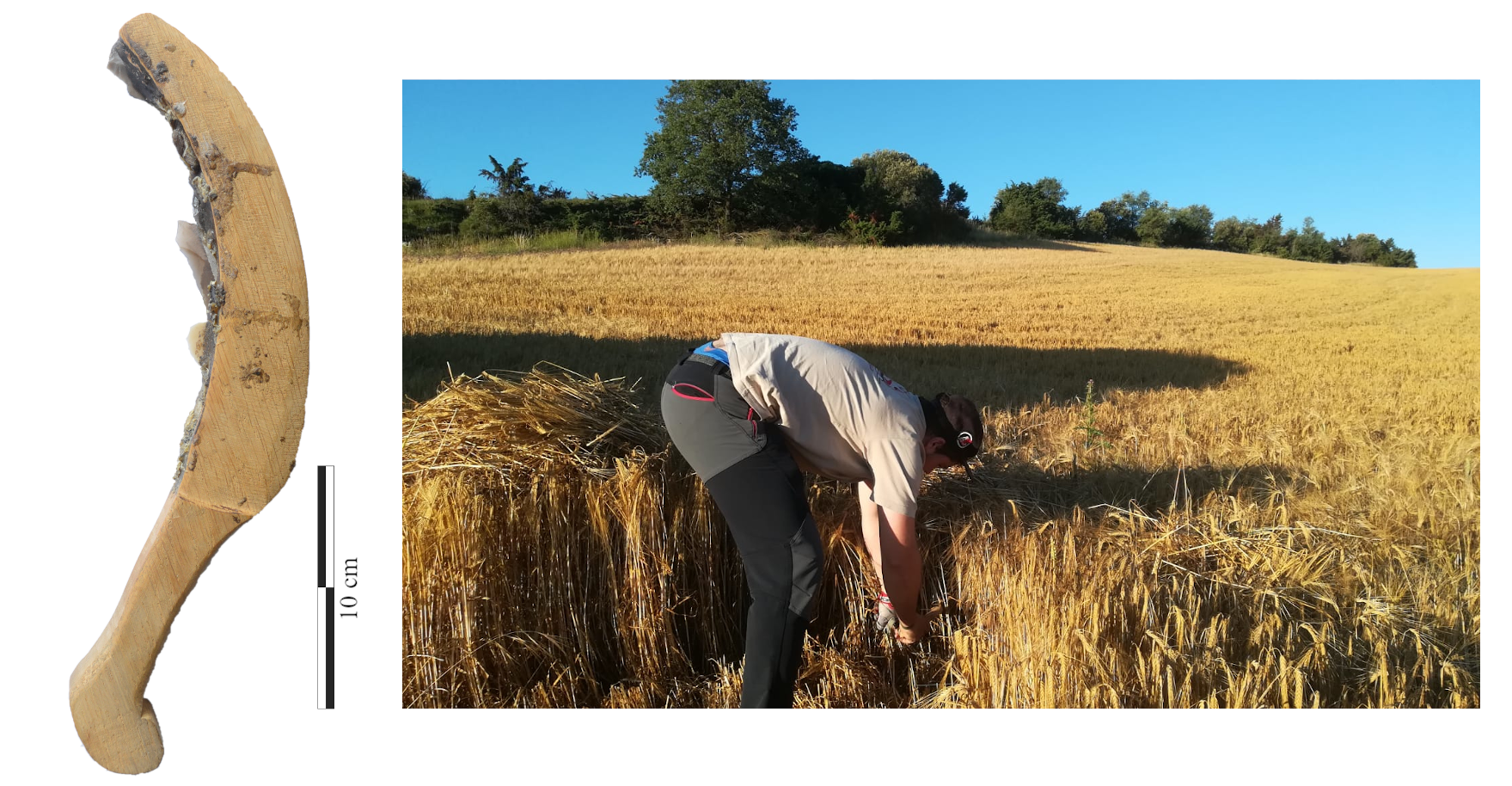


Figure S2-3 Experimental sickle and harvested field of *Hordeum* *vulgare* (Spain).

*Harvesting reeds.* Green reeds (*Phragmites australis*) have been harvested in July 2016 in northern France (Eure-et-Loir) with an experimental straight sickle. Approximately 5m2 corresponding to 1000 stems have been cut for 2 hours. Three unretouched fine-grained flint blades (Fécamp – France) were fixed in a parallel way with bitumen in a wooden haft. Stems have been harvested with a low height cutting (ca. 10 cm from the ground), with some contact between the sickle and leaves of the stems.


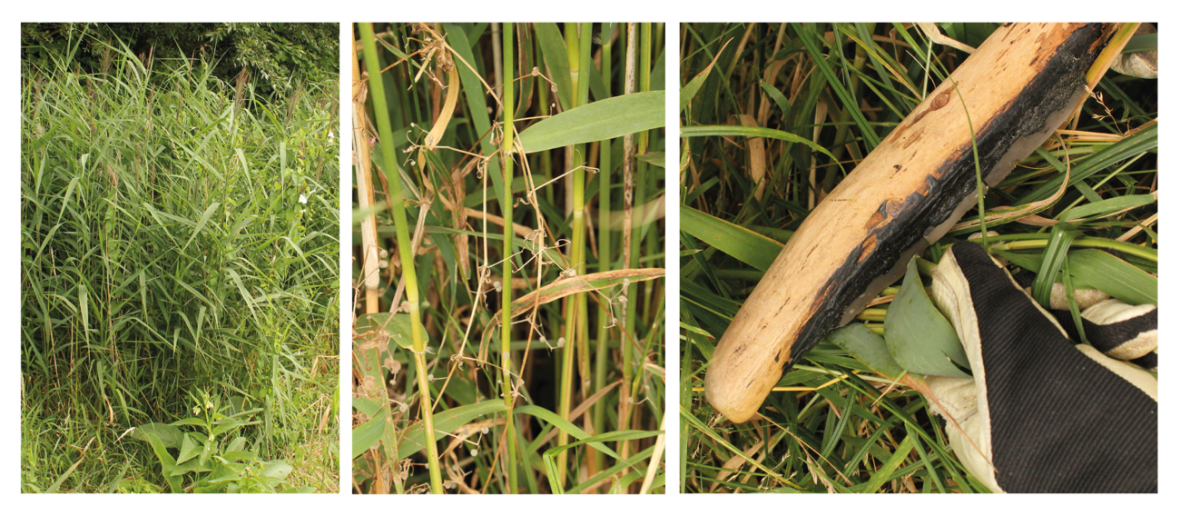


Figure S2-4. Experimental sickle and harvested field of green reeds (*Phragmites australis*).

*Harvesting grasses.* Different grasses belonging to the *Festuca* and *Juncus* genus were harvested in 2011 in the Pyrenean area (Aigüestortes i Estany de Sant Maurici National Park). Two stone tools on fine-grained flint were used, both hand-held. Each tool was used to cut grasses for one hour of working time, cutting at a low height, with an occasional contact with soil.

**
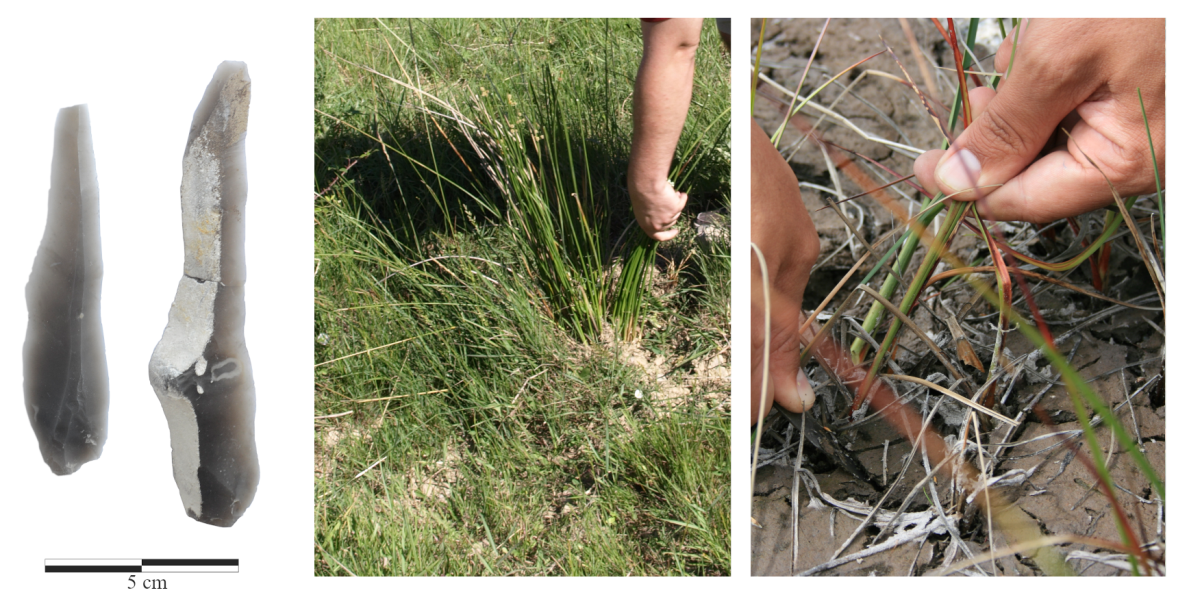
**

Figure S2-5. Experimental tools and harvested grasses (*Juncus* sp*.*).

*Harvesting Triticum dicoccum.* Emmer wheat was harvested in August 2021 in Asturias (Spain), near the village of Quinzanas. *Triticum dicoccum* was harvested in a ripe state with two sickles: one cutting low, near the ground, the other cutting high, just under the spikelet. Both tools were used for 6 hours. Sickles were characterized by a straight handle with four lithic inserts each. Lithic inserts have been shaped by abrupt direct retouch on bladelets of different varieties of fine-grained flints (i.e. Gargano – Italy; Fécamp – France) and two obsidian blades from Caucasus.


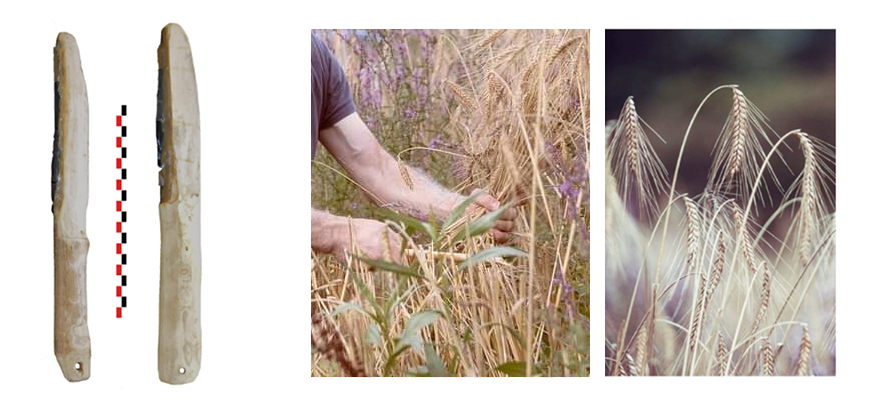


Figure S2-6. Experimental sickles (left: high height cutting; right: low height cutting) and harvested field of *Triticum dicoccum* (Spain).

*Harvesting Triticum spelta.* Spelta wheat was harvested in a ripe state in August 2021, in Asturias (Spain), near the village of Quinzanas. Two curved sickles were used to harvest *Triticum spelta*. One was used for 12 hours, with a low cutting near the ground, and the other for 16 hours, cutting high, just under the spikelet. Both sickles had five inserts, four on fine-grained flints of different varieties (i.e. Gargano and Apennines flints – Italy; Fécamp flint – France), and one made of obsidian from Caucasus.


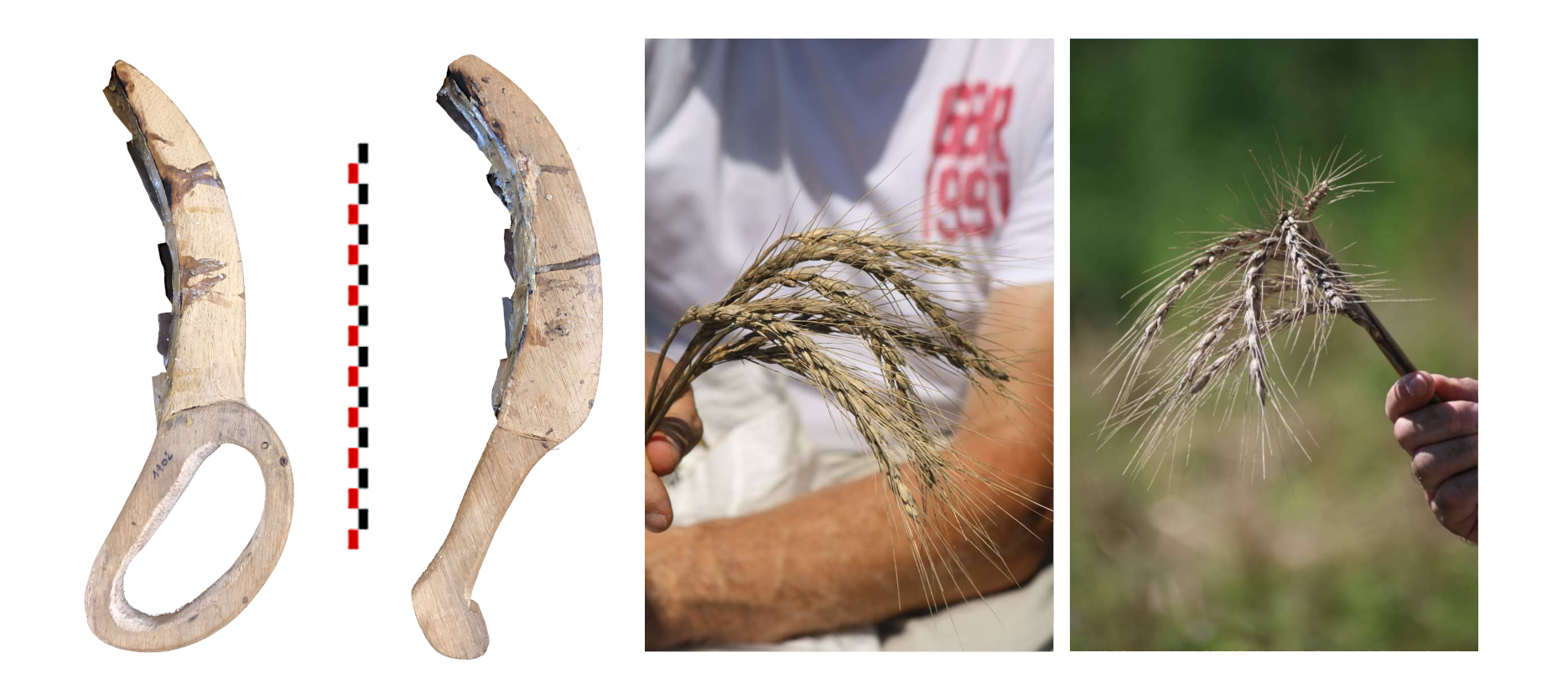


Figure S2-7. Experimental sickles (left: low height cutting; right: high height cutting) and harvested *Triticum spelta* (Spain).
