# Supplementary material for: Multiproxy study of 7500-year-old wooden sickles from the Lakeshore Village of La Marmotta, Italy
Source: Sci Rep. 2022 Sep 2;12:14976. doi: 10.1038/s41598-022-18597-8 (PMC9440057; doi:10.1038/s41598-022-18597-8)
Supplement: Supplementary file 3 — Supplementary Information 3. [file 41598_2022_18597_MOESM3_ESM.docx]

# S3. Use-wear and microtexture analysis through confocal microscopy.

**Test1 – Testing the type of harvested plants**

*Data sampling.* To test the type of harvested plants, archaeological sickles were compared with a selection of experimental tools used on different cereal and non-cereal plants (see Supplementary material S2 for more details on the experiments). A total of 10 experimental tools were used as reference framework, including six inserts from three wooden/antler sickles used for harvesting a diversity of domesticated crops, and four unhafted lithic tools used to cut different wild plants (Supplementary Figures S3-1-7). A totality of 10 lithic tools were selected to create a reference experimental framework to classify archaeological tools (Supplementary Table S3-1). For what concerns the three archaeological sickles, only inserts showing the most developed traces have been selected for quantitative analysis: five inserts for sickle 44297, three inserts for sickle 7186, and seven inserts for sickle 23001 (Supplementary Table S3-1).

The number of selected areas for each tool (both experimental and archaeological ones) varies from 2 to 17 depending on the extension of the use-wear on the stone tool surface (Supplementary Table S3-1). A totality of 271 zones have been measured with S Neox SensoSCAN software, including both archaeological and experimental tools. Afterwards, between 1 and 5 subareas showing well-developed use-wear have been sampled for each area. A total of 551 subareas were sampled, 164 for experimental tools and 387 for the archaeological ones (Supplementary Table S3-1). All subareas were processed with Mountains7 software from Digital Surf. For each one of them a leveling operator using the least squares (LS) plane method was used to correct the lack of horizontality. After, a form removal operator was used to remove the base form comprising the surface. Spatial filtering is then applied to isolate the roughness components of the surfaces using a Gaussian filter with a 0.08 mm cut-off. Finally, 45 different texture parameters have been extracted for each subarea. 42 parameters included in the ISO 25178 standard were selected, and in addition three parameters measuring the furrows contained in each surface, measuring their maximum depth, mean depth and mean density.

| **TYPE** | **TOOL** | **N° ZONES** | | **N° SUBAREAS** |
| --- | --- | --- | --- | --- |
| EXP | Triticum monococcum-EXP1 | | 11 | 33 |
| EXP | Triticum aestivum-P5 | | 16 | 14 |
| EXP | Triticum aestivum-P7 | | 15 | 4 |
| EXP | Triticum aestivum-P8 | | 17 | 7 |
| EXP | Hordeum vulgare-P3 | | 12 | 5 |
| EXP | Hordeum vulgare-P6 | 16 | | 20 |
| EXP | Grass-EXP1 | 16 | | 31 |
| EXP | Grass-EXP2 | 8 | | 15 |
| EXP | Reeds-EXP88 | 8 | | 21 |
| EXP | Reeds-EXP89 | 10 | | 14 |
| ARCH | 44297-3 | 8 | | 45 |
| ARCH | 44297-5 | 9 | | 21 |
| ARCH | 44297-6 | 7 | | 8 |
| ARCH | 44297-7 | 12 | | 20 |
| ARCH | 44297-8 | 17 | | 22 |
| ARCH | 7186-1 | 7 | | 19 |
| ARCH | 7186-2 | 11 | | 45 |
| ARCH | 7186-3 | 12 | | 33 |
| ARCH | 23001-1 | 17 | | 60 |
| ARCH | 23001-3 | 8 | | 24 |
| ARCH | 23001-4 | 12 | | 17 |
| ARCH | 23001-5 | 12 | | 41 |
| ARCH | 23001-6 | 2 | | 4 |
| ARCH | 23001-7 | 9 | | 19 |
| ARCH | 23001-9 | 4 | | 9 |
| Total | | 276 | | 551 |

Supplementary Table S3-1. Number of sampled areas and subareas for each tool used for test1.


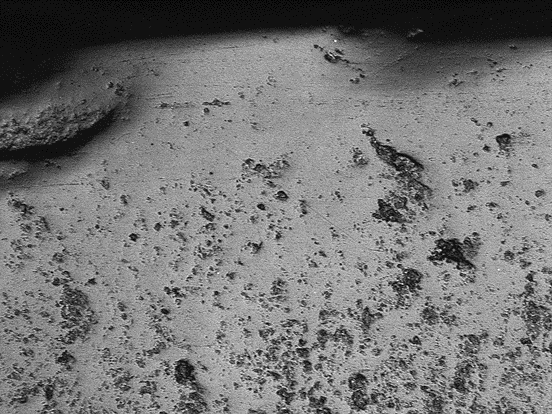


Supplementary Figure S3-1. Harvesting Triticum monococcum in France, insert 1, 10h, 200x

*
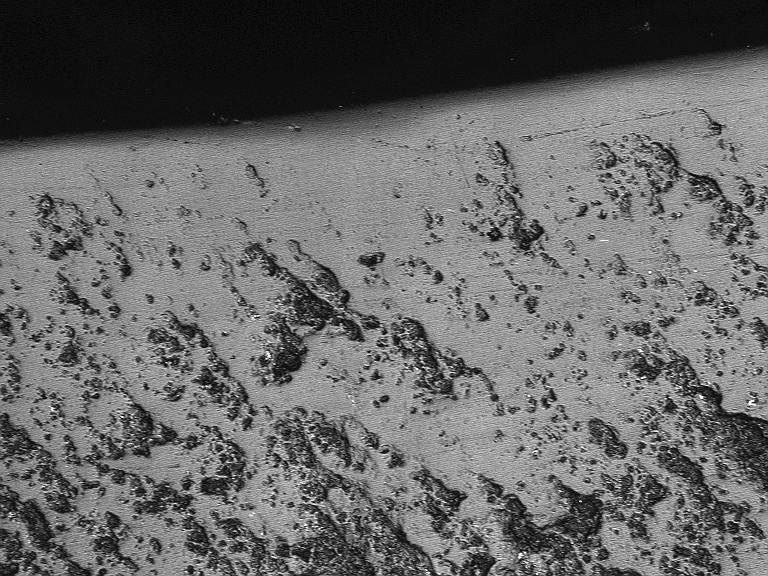
*

Supplementary Figure S3-2. Harvesting Triticum aestivum in Tuscany, insert 7, 15h, 200x

*
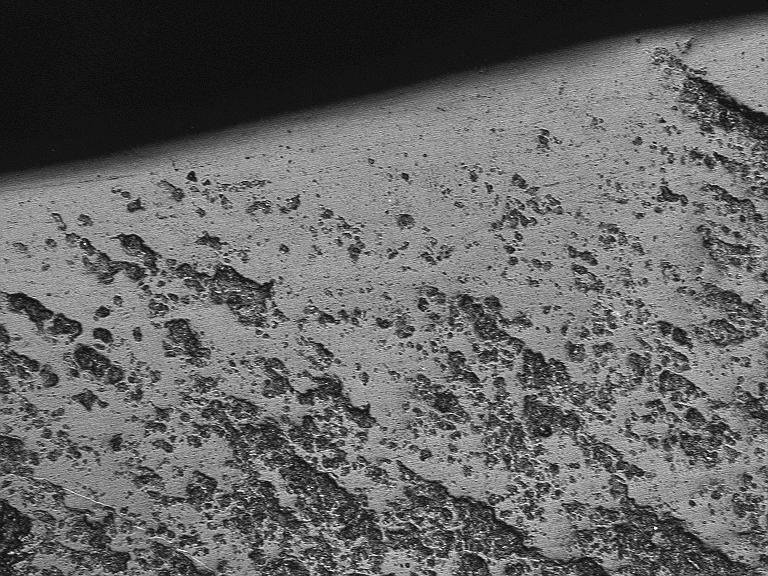
*

Supplementary Figure S3-3. Harvesting Triticum aestivum in Tuscany, insert 5, 15h, 200x

*
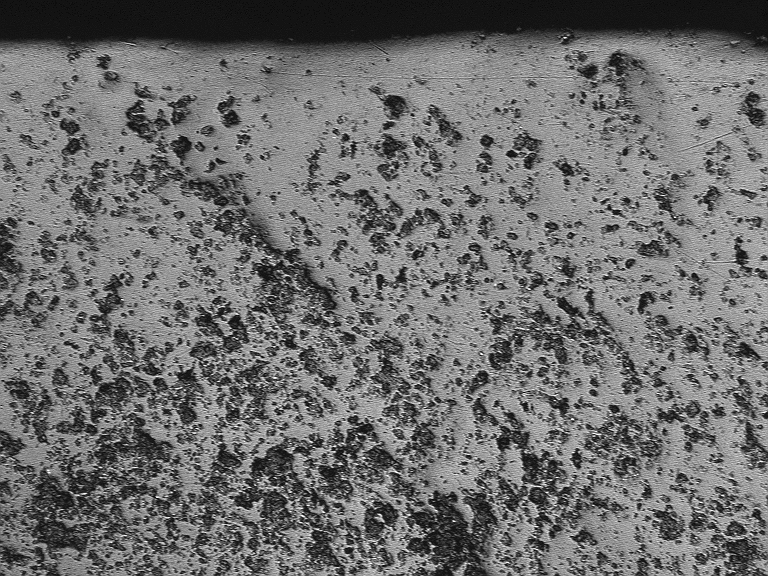
*

Supplementary Figure S3-4. Harvesting Hordeum vulgare in Spain, insert 6, 8h, 200x


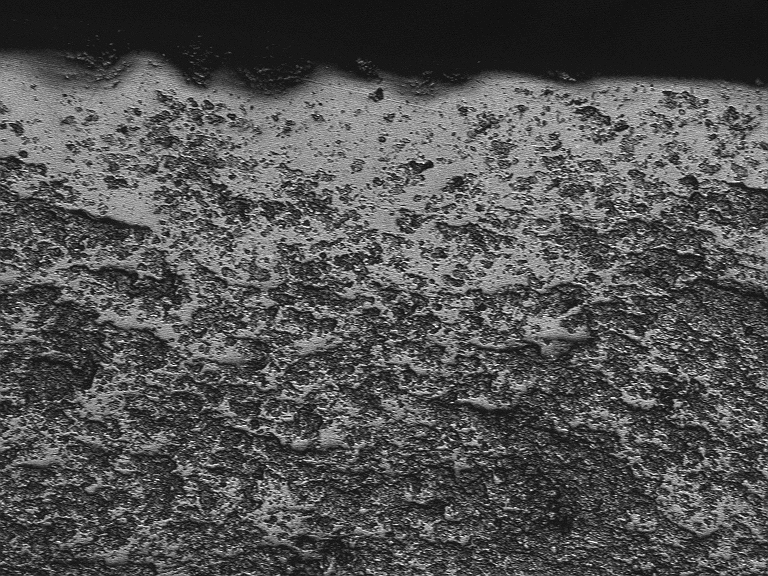


Supplementary Figure S3-5. Harvesting Hordeum vulgare in Spain, insert 3, 8h, 200x


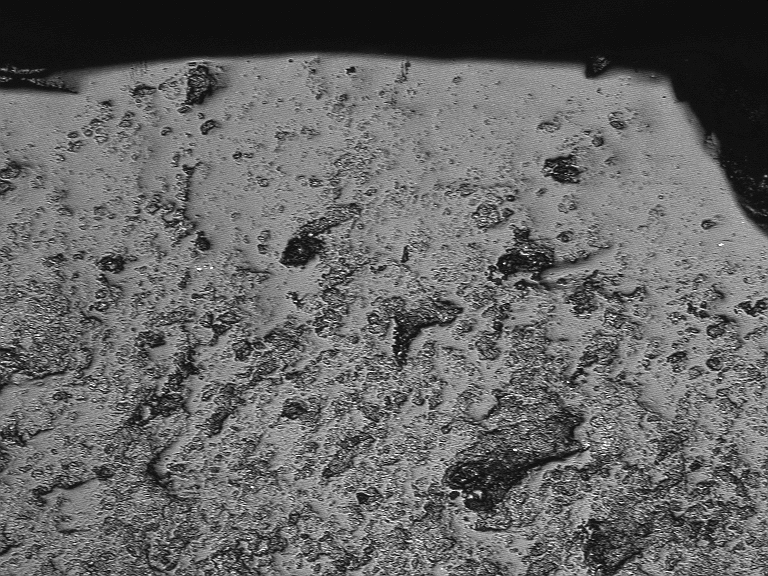


Supplementary Figure S3-6. Harvesting Phragmites australis in France, tool 88, 2h, 200x


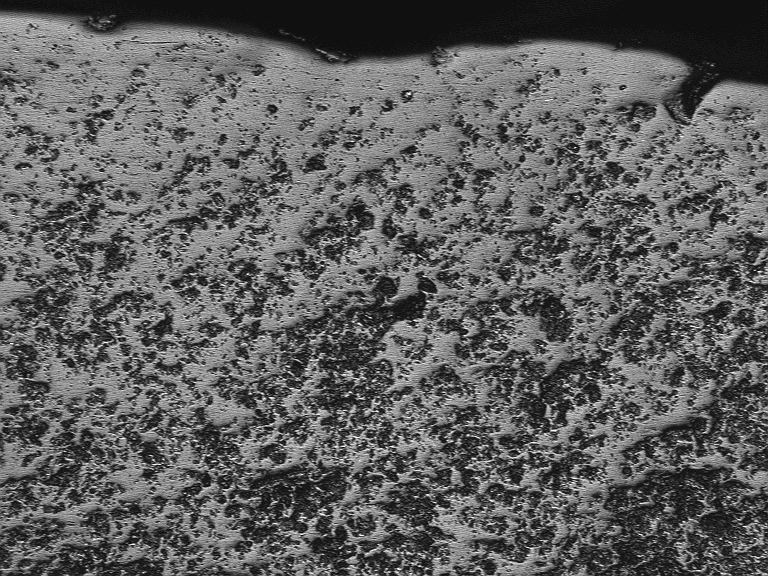


Supplementary Figure S3-7. Harvesting Juncus sp. in Spain, tool 1, 1h, 200x

*Statistical analysis.* Statistical analysis has been carried out with SPSS v.12. First, in order to considerably reduce the number of texture parameters and select the most relevant predictors a stepwise discriminant analysis using Mahalanobis distance has been carried out. The stepwise method began with a model that does not include any predictor. At each step, a predictor is added to the model using F probability as cutoff (F=0.5). Of all parameters that have been entered in the analysis, only parameters with values greater than 0.400 in the structure matrix have been retained. As results, seven predictors have been selected:

- *Height parameters*: *Sq*, the square root mean height;
- *Functional volume parameters*: *Vvv*, valley void volume, which represents the void volume of dales at the areal material ratio p =80%;
- *Functional parameters*: *Sk*, core roughness depth (peak-to-valley), which is the difference of heights at areal material ratio p =80%; specifically, it is a value obtained by subtracting the minimum height from the maximum height of the core surface of the surface with the predominant peaks and valleys removed.
- *Hybrid parameters*: *Sdr*, developed interfacial area ratio, which indicates the complexity of the surface thanks to the comparison of the curvilinear surface and the support surface; *Sds*, density of summits, which counts the number of summits per unit area of the surface; *Ssc*, mean summit curvature, indicates the mean form of peaks and valleys.
- *Areal feature parameters*: *Spd*, density of peaks, which represents the number of peaks per unit area.

Quadratic discriminant function analysis was therefore run using these seven predictors, building a predictive model for the three groups: domesticated cereals, reeds and grasses. The results of this blind classification indicate that the 79.9% of the cases are correctly classified into one of the three groups (Supplementary Figure S3-8 and Supplementary Table S3-2).


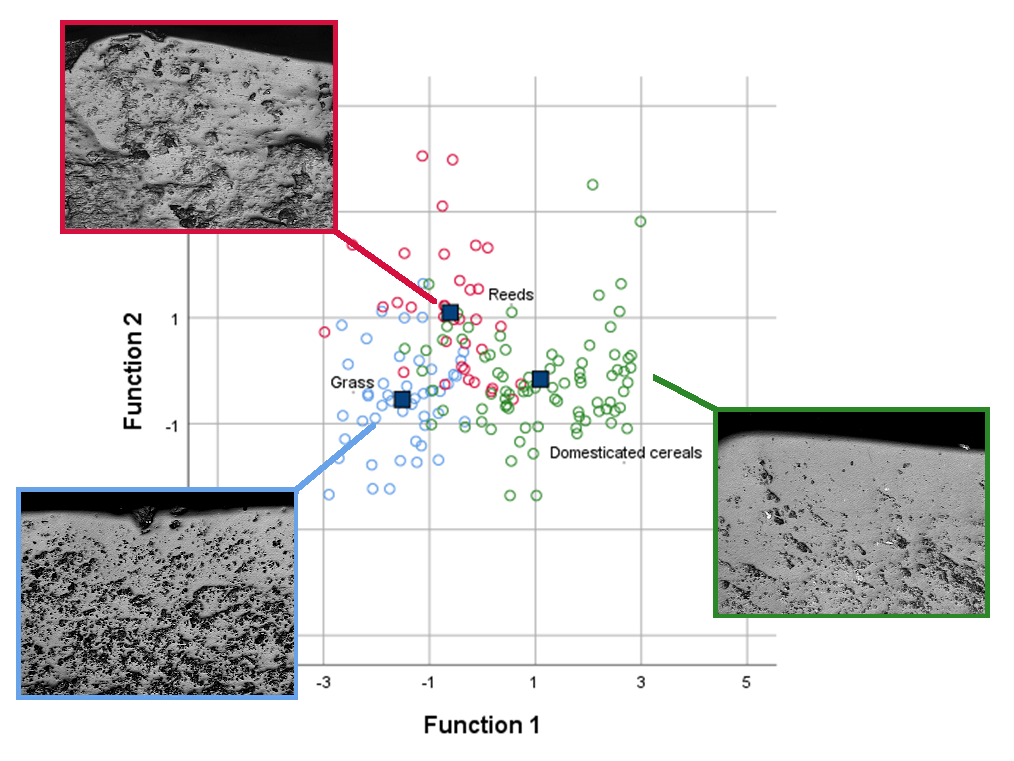


Supplementary Figure S3-8. Classification results from the Canonical Discriminant Analysis for the three groups. Blue squares indicate the centroid of each experimental group. Micrographs have been taken from the experimental tools to exemplify the appearance of use-wear for each class.

| **Classification Results** | | | | | |
| --- | --- | --- | --- | --- | --- |
|  | | GRASS | REEDS | DOM | Tot |
| ∑ | GRASS | 39 | 5 | 2 | 46 |
|  | REEDS | 3 | 22 | 10 | 35 |
|  | DOM | 6 | 7 | 70 | 83 |
| % | GRASS | 84,8 | 10,9 | 4,3 | 100% |
|  | REEDS | 8,6 | 62,9 | 28,6 | 100% |
|  | DOM | 7,2 | 8,4 | 84,3 | 100% |

Supplementary Table S3-2 Classification results from the Canonical Discriminant Analysis for the three groups.

Domesticated cereals show less rough (lower values of *Sq*) and complex (lower *Sdr*) textures, with a lower density of peaks (lower *Spd*) and a thinner core (lower *Sk*). Lower values of *Vvv* indicate that valley structures were smaller in volume in respect to Grass and Reeds, while lower values of *Ssc* denotes more rounded of peaks and valleys. Conversely, higher values of *Sds*, as shown by domesticated cereals, are expected in more polished specimens in respect to rougher ones. *Sds* is indeed negatively correlated with area roughness parameters such as *Sq,* *Sk*, and *Ssc* and therefore higher value of *Sds* also indicates smoother surfaces[^1^](#_Dimkovski,_Z.,_Anderberg,).

At this point, subareas from the archaeological specimens have been blindly classified in one of the three groups (Supplementary Figure S3-9). An individual classification for each subarea has been obtained, with the 95.1% of archaeological areas attributed to domesticated cereals.


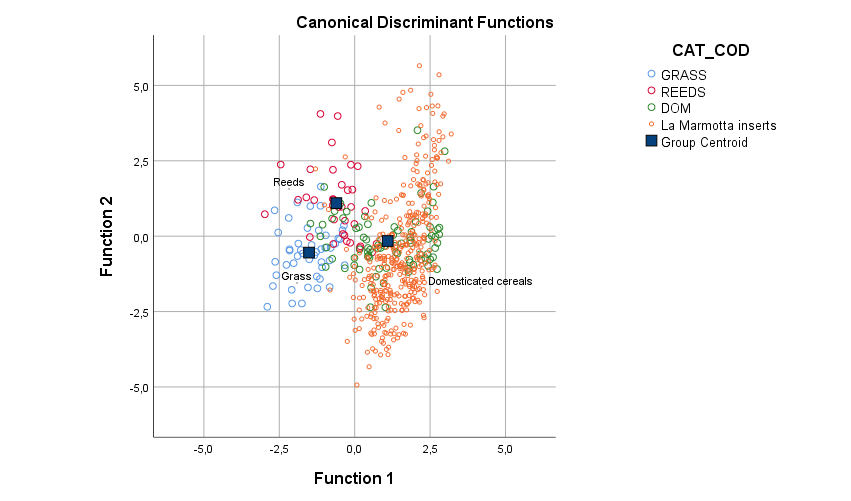


Supplementary Figure S3-9. Classification results from the Canonical Discriminant Analysis including subareas from La Marmotta sickles (orange points). Blue squares indicate the centroid of each experimental group. In table, the predicted group for each stone insert are reported.

However, to correctly classified each insert, all subareas belonging to the same stone tools have been grouped together (Supplementary Table S3-3). As results, 14 of the 15 analyzed inserts are classified as having harvested domesticated cereals (more than 60% of subareas classified into one of the three groups). Only one insert (23001-9) is classified as having cut reeds. However, this latter insert shows a very little development of the wears (also see Figure 4, 9). Different explications are possible. This insert has been possibly added later to the sickle, and, therefore, the use-wear traces on its surfaces are testifying a last episode of harvesting of non-cereal plants that is not detectable on the other inserts of sickle Nº 23001. However, we should also keep in mind that the low development of the use-wear is a factor that can affect the classification capacity, as also suggested from the experimental study of Ibáñez & Mazzucco^2^.

|  | | **Predicted group for each insert** | | | | |
| --- | --- | --- | --- | --- | --- | --- |
|  |  | GRASS | REEDS | | DOM | |
| 44297-3 | |  | |  | | **100,0%** |
| 44297-5 | |  | | 4,8% | | **95,2%** |
| 44297-6 | |  | | 37,5% | | **62,5%** |
| 44297-7 | |  | |  | | **100,0%** |
| 44297-8 | |  | |  | | **100,0%** |
| 7186-1 | | 15,8% | |  | | **84,2%** |
| 7186-2 | |  | | 8,9% | | **91,1%** |
| 7186-3 | |  | |  | | **100,0%** |
| 23001-1 | |  | |  | | **100,0%** |
| 23001-3 | |  | |  | | **100,0%** |
| 23001-4 | |  | |  | | **100,0%** |
| 23001-5 | |  | |  | | **100,0%** |
| 23001-6 | |  | |  | | **100,0%** |
| 23001-7 | |  | |  | | **100,0%** |
| 23001-9 | |  | | **88,9%** | | 11,1% |
| Tot | 8,2% | | 9,0% | | 82,8% | |
|  |  | |  | |  | |

Supplementary Table S3-3. Predicted groups from the Canonical Discriminant Analysis including all subareas from La Marmotta sickles. All subareas belonging to the same insert are grouped together.

**Test2 – Testing low versus high height harvesting**

*Data sampling.* A second test has been carried out to classify the zones previously sampled on the archaeological tools with a new experimental reference framework: four sickles used to cut two species of wheat (*Triticum dicoccum* and *Triticum spelta*), performing for each sickle both a low and a high height harvesting (see Supplementary Material S2). This test was interesting to discriminate whether archaeological sickles were used for ears harvesting, cutting high, or for cutting the straw low, near the ground.

As for the test1, for each experimental and archaeological sickle only inserts showing the most developed traces have been selected (Supplementary Table S3-4). The number of selected areas varies from 12 to 17 depending on the extension of the use-wear on the stone tool surface. The selected areas for the archaeological tools are the same of test1. As result, a totality of 269 areas have been measured with S Neox SensoSCAN software, including both archaeological and experimental tools. Afterwards, between 1 and 5 subareas showing well-developed use-wear have been sampled for each area. 111 subareas were sampled from the experimental tools that, summed to the 387 previously sampled from the archaeological tools, give a total of 498 subzones (Supplementary Table S3-4).

All subareas were processed with Mountains7 software from Digital Surf. For each one of them a leveling operator using the least squares (LS) plane method was used to correct the lack of horizontality. After, a form removal operator was used to remove the base form comprising the surface. Spatial filtering is then applied to isolate the roughness components of the surfaces using a Gaussian filter with a 0.08 mm cut-off. Finally, 13 different texture parameters have been extracted for each subarea. 12 parameters included in the ISO 25178 standard were selected, and in addition one parameter measuring the mean deep of the furrows contained in each surface.

| **TYPE** | **TOOL** | **N° ZONES** | | **N° SUBAREAS** |
| --- | --- | --- | --- | --- |
| EXP | Triticum spelta-high-E3 | | 17 | 23 |
| EXP | Triticum spelta-low-E2 | | 13 | 39 |
| EXP | Triticum dicoccum-high-E2 | | 12 | 26 |
| EXP | Triticum dicoccum-low-E1 | | 15 | 23 |
| ARCH | 44297-3 | | 8 | 45 |
| ARCH | 44297-5 | | 9 | 21 |
| ARCH | 44297-6 | | 7 | 8 |
| ARCH | 44297-7 | 12 | | 20 |
| ARCH | 44297-8 | 17 | | 22 |
| ARCH | 7186-1 | 7 | | 19 |
| ARCH | 7186-2 | 11 | | 45 |
| ARCH | 7186-3 | 12 | | 33 |
| ARCH | 23001-1 | 17 | | 60 |
| ARCH | 23001-3 | 8 | | 24 |
| ARCH | 23001-4 | 12 | | 17 |
| ARCH | 23001-5 | 12 | | 41 |
| ARCH | 23001-6 | 2 | | 4 |
| ARCH | 23001-7 | 9 | | 19 |
| ARCH | 23001-9 | 4 | | 9 |
| Total |  | 204 | | 498 |

Supplementary Table S3-4. Number of sampled areas and subareas for each tool used for test2.

*Statistical analysis.* Statistical analysis has been carried out with SPSS v.12. First, to considerably reduce the number of texture parameters and select the most relevant predictors a stepwise discriminant analysis using Mahalanobis distance has been carried out. The stepwise method began with a model that does not include any predictor. At each step, a predictor is added to the model using F probability as cutoff (F=0.5). Of all parameters that have been entered in the analysis, only parameters with values greater than 0.400 in the structure matrix have been retained. As results, only two predictors have been selected:

- *Functional parameters: Smc*, inverse areal material ratio; *Sxp*, peak extreme height.

Quadratic discriminant function analysis was run using only these two predictors, building a predictive model for the two groups: low height harvesting and high height harvesting. The results of this blind classification indicate that the 90.1% of the cases are correctly classified (Supplementary Table S3-5).

| **Classification Results** | | | | |
| --- | --- | --- | --- | --- |
|  | | HIGH | LOW | Tot |
| ∑ | HIGH | 38 | 11 | 49 |
|  | LOW | 0 | 62 | 62 |
| % | HIGH | 77,6 | 22,4 | 100% |
|  | LOW | ,0 | 100,0 | 100% |
|  |  |  |  |  |

Supplementary Table S3-5 Classification results from the Canonical Discriminant Analysis for the three groups.

Cereals harvested high show higher values for both parameters (*Sxp* and *Smc*) in respect to tools used for low height cutting (Supplementary Figure S3-10). This suggest that low cutting harvesting tends to produce flatter and less rough surfaces, possibly because the wear process is enhanced by the including of abrasive particles from the soil/ground. Such a difference between low and heigh harvesting are clear as well at a visual inspection of the use-wear wear, with polish from low cutting harvesting that appear flatter and more striated (Supplementary Figure S3-11).


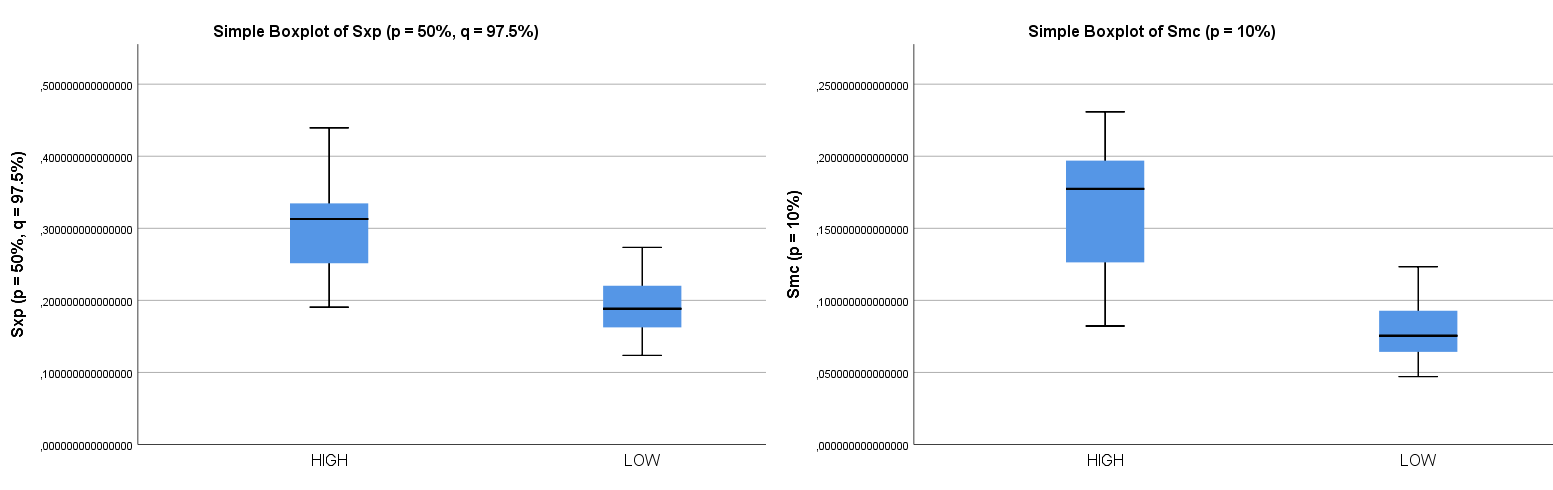


Supplementary Figure S3-10. Boxplot with values of *Sxp* and *Smc* for High and Low height harvesting.


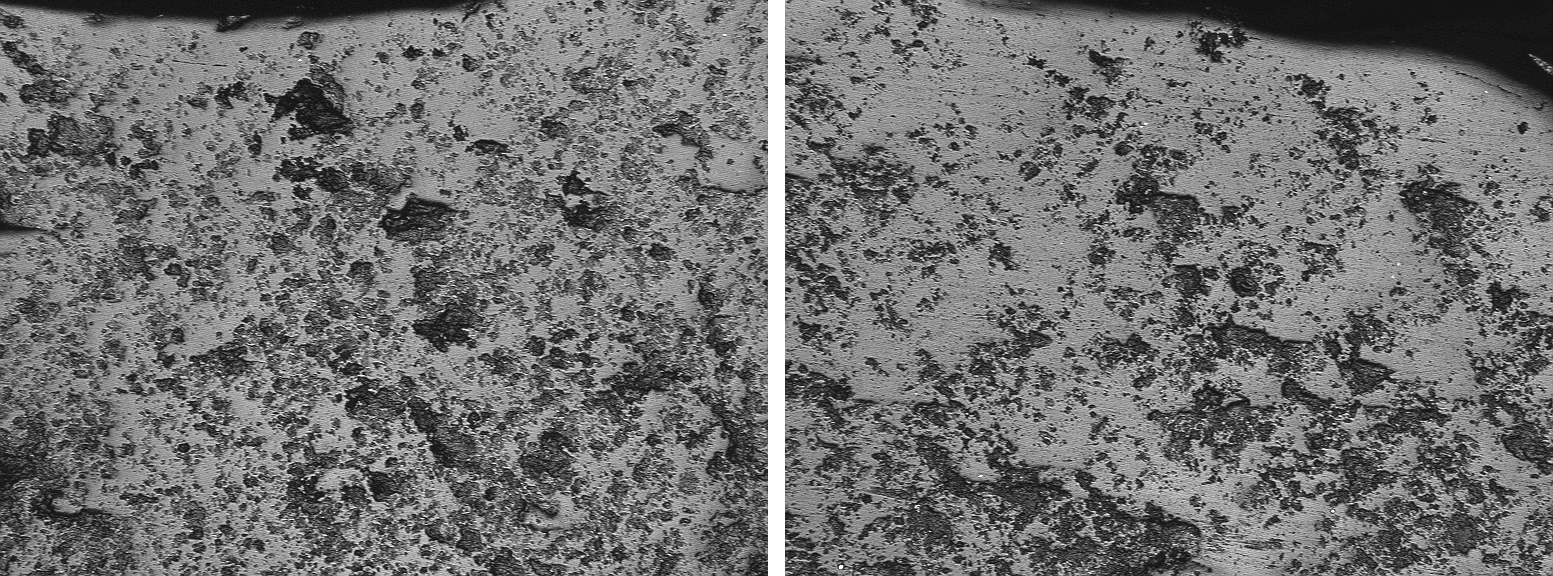


Supplementary Figure S3-11. Left: low height harvesting, 200x (*Triticum dicoccum,* 6 hours); Right: high height harvesting, 200x (*Triticum dicoccum,* 6 hours).

At this point, subareas from the archaeological specimens have been blindly classified in one of the three groups. An individual classification for each subarea has been obtained, with the 93.3% of areas attributed to low height harvesting (Supplementary Figure S3-12). To test the harvesting method of each individual sickle, we grouped subareas from the same sickle together (Supplementary Table S3-6). As results, all three sickles are classified as having harvested low with over 90% of subareas classified as such.

| **Predicted Group for La Marmotta’s sickles** | | | |
| --- | --- | --- | --- |
|  | HIGH | LOW | Tot |
| No. 44297 | 9,4% | 90,6% | 100% |
| No. 7186 | 5,2% | 94,8% | 100% |
| No. 23001 | 5,7% | 94,3% | 100% |
| Tot | 6,7% | 93,3% | 100% |

**
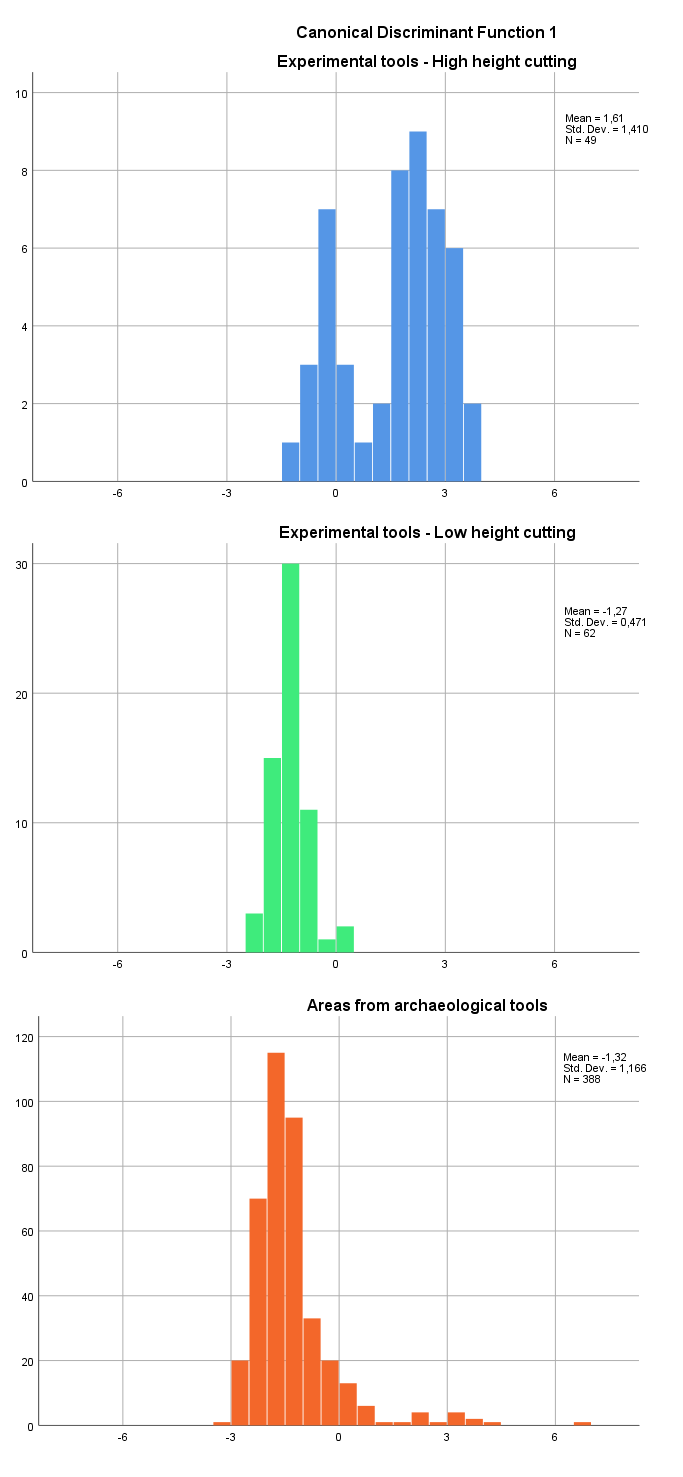
**Supplementary Table S3-6. Number of sampled areas and subareas for each sickle, test2.

Supplementary Figure S3-12. Discriminant scores for Function 1 for experimental and archaeological tools.

# References

## Dimkovski, Z., Anderberg, C., Ohlsson, R. & Rosén, B.-G. Characterisation of Cylinder Liner Honing Textures for Production Control. in Characterisation of areal surface texture (ed. Leach, R.), 281-302 (London, Springer, 2013).

## Ibáñez, J. J., Mazzucco N. Quantitative use-wear analysis of stone tools: Measuring how the intensity of use affects the identification of the worked material. PLoS ONE **16(9)**: e0257266 (2020). https://doi.org/10.1371/journal.pone.0257266
