# Supplementary material for: Multiproxy study of 7500-year-old wooden sickles from the Lakeshore Village of La Marmotta, Italy
Source: Sci Rep. 2022 Sep 2;12:14976. doi: 10.1038/s41598-022-18597-8 (PMC9440057; doi:10.1038/s41598-022-18597-8)
Supplement: Supplementary file 4 — Supplementary Information 4. [file 41598_2022_18597_MOESM4_ESM.docx]

# S4. Organic residue extraction, GC and GC-MS analyses.

GC and GC-MS analyses were performed on an Agilent Technologies 7890B GC System series chromatograph including Agilent Technologies Capillary Flow-Technology Three-Way Splitter Kit coupled to an Agilent Technologies 5977A MSD and FID. The analyses were carried out using helium as a carrier gas, with a split/splitless injection system (Gerstel Multi-Purpose Sampler and Gerstel Cold-Injection-System 4), operating in the splitless mode with a purge flow of 3.0 ml min–1 and a constant pressure at the head of the column of 8.6667 psi. Samples were analysed using an Agilent J&W DB-5HT-column (15 m × 0.32 mm i.d.; 0.1 μm film thickness) and divided in two equal parts using 0.18 mm non-coated, deactivated silica capillary columns (0.66 m splitter-column to FID/ 1.52 m splitter-column to MSD) with the ThreeWay Splitter Kit. The inlet temperature was ramped from 30˚C to 240˚C at 12˚C s-1 (held isothermally for 5 min) and then increased to 350˚C at 12˚C s-1 (held isothermally for 10 min). The temperature of the oven was set at 50˚C for 1 min followed by an increase to 100˚C at 15˚C min–1, then to 240˚C at 4˚C min–1 and to 380˚C at 20˚C min–1 (held isothermally for 7 min). Mass spectra were acquired using electron ionization at 70 eV and obtained by scanning between m/z 50–950 in 1.562 s. The interface and the ion source temperatures were 300˚C and 280˚C, respectively. The temperature of the FID detector was fixed at 340˚C. Mass spectra were matched using published data^1-6^ and the National Institute of Standards and Technology (NIST) library, 2014 edition.

Molecular analyses of organic residues adhering to the 3 sickles show the presence of abietane diterpenoids (e.g., abietic, dehydroabietic, 7-oxo-dehydroabietic and abieta-6,8,11,13-tetraen-18-oic acids) and Pimarane diterpenoids (e.g., pimaric and isopimaric acid) which are characteristic of conifer by-products^1^, and even Pinaceae by-products in archaeological contexts^2,3^. Among the very limited data on the use of conifer by-products throughout prehistory^4-13^, it is not always possible to distinguish the Genus exploited or the type of material (tar, resin etc.) due to the degradation of molecular assemblages (e.g.,^4-12^). However, in contexts more favourable for the preservation of residues, characteristic markers can specify the Genus. This is the case of the epi-manool markers of spruce and Larch^10-12^ or the assemblage of pimaric, isopimaric and abietic acids associated with pine^6,10,13^. The presence of the latter, as well as Seco- dehydroabietic acid (α and/or β) in the adhesives of one sickle (187882), supports a pine origin^10,13^. As for most archaeological or aged resins, the identification of 7-oxo-dehydroabietic, abieta-6,8,11,13-tetraen-18-oic and 7-hydroxy dehydroabietic acids are related to various diagenetic processes of resin alteration (aromatisation or oxidation) which could be formed by anthropic or natural transformation of the resin^6^. The absence of heating markers (hydrocarbon/aromatic or methylate diterpenes) does not suggest the presence of pitch or tar, but rather a resin^14^.

Figure S4-1. GC-MS spectrum for La Marmotta samples. Pinaceae resin. 1: seco-dehydroabietic acid, β isomer; 2: seco-dehydroabietic acid, α isomer; 3: pimaric acid; 4: isopimaric acid; 5: abieta-6,8,11,13-tetraen-18-oic acid; 6: dehydroabietic acid; 7: abietic acid; 8: hydroxy dehydroabietic acid isomer; 9: 7-hydroxy dehydroabietic acid x: pimaranoic/abietaneoic acid isomers. *Restauration material (polyethylene glycol)


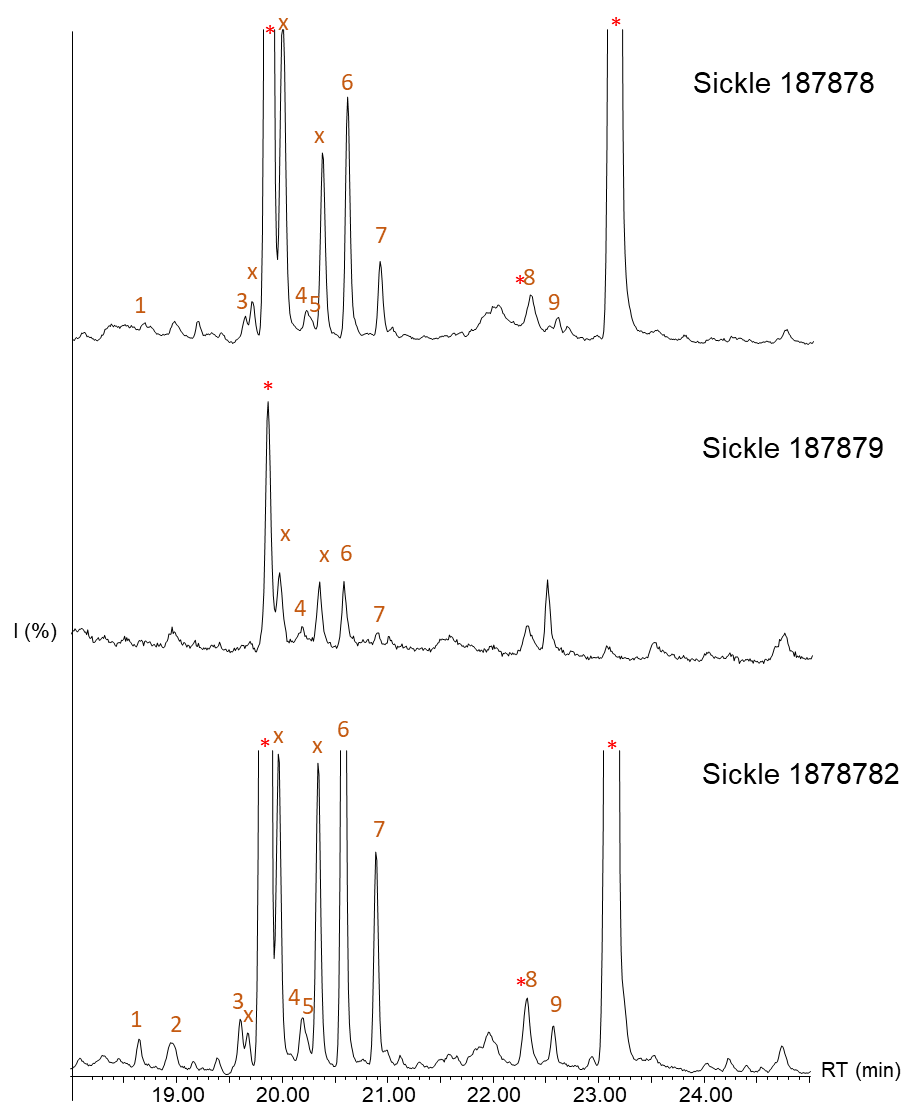


**Sickle – 44297**

**Sickle – 7186**

**Sickle – 23001**

#

# References

### van den Berg, K. J., Boon, J. J., Pastorova, I. & Spetter, L. F. M. Mass spectrometric methodology for the analysis of highly oxidized diterpenoid acids in Old Master paintings. Journal of Mass Spectrometry 35, 512-533, doi:10.1002/(sici)1096-9888(200004)35:4<512::aid-jms963>3.0.co;2-3 (2000).

### Mills, J. S. & white, R. Natural resins of art and archaeology their sources, chemestry, and identification. Studies in Conservation 22, 12-31, doi:doi:10.1179/sic.1977.003 (1977).

### Mills, J. S. & White, R. The organic chemistry of museum objects. second edn (Butterworth-Heineman, (1994).

### Heron, C., Evershed, R. P., Chapman, B. & Pollard, A. M. in P. Budd, B. Chapman, C. Jackson, R. Janaway & B. Ottaway (eds.) Archaeological Sciences 1989. Proceedings of a Conference on the Application of Scientific Techniques to Archaeology: 325-31. (Oxford: Oxbow, Bradford, 1991).

### Evans, K. & Heron, C. Glue, Disinfectant and Chewing Gum: Natural Products Chemistry in Archaeology. Chemistry and Industry june 21, 446-449 (1993).

### Regert, M. Investigating the history of prehistoric glues by gas chromatography-mass spectrometry. journal of separation science 27, 244-254 (2004).

### Urem-Kotsou, D., Stern, B., Heron, C. & Kotsakis, K. Birch bark tar at Neolithic Makriyalos, Greece. Antiquity 76, 962-967 (2002).

### Mitkidou, S. et al. Organic residue analysis of Neolithic Pottery from North Greece. Microchimica Acta 160, 493-498 (2008).

### Urem-Kotsou, D., Mitkidou, S., Dimitrakoudi, E., Kokkinos, N. & Ntinou, M. Following their tears: Production and use of plant exudates in the Neolithic of North Aegean and the Balkans. Quaternary International 496, 68-79, doi:10.1016/j.quaint.2018.10.027 (2018).

### Helwig, K., Monahan, V. & Poulin, J. The identification of hafting adhesive on a slotted antler point from a southwest Yukon ice patch. American Antiquity 73, 279-288 (2008).

### Helwig, K., Monahan, V., Poulina, J. & Andrews, T. D. Ancient projectile weapons from ice Patches in Northwestern Canada: identification of resin and compound resin-ochre hafting adhesives. Journal of Archaeological Science 41, 655-665 (2014).

### Degano, I. et al. Hafting of Middle Paleolithic tools in Latium (central Italy): New data from Fossellone and Sant’Agostino caves. PLOS ONE 14, e0213473, doi:10.1371/journal.pone.0213473 (2019).

### Rageot, M. et al. Management systems of adhesive materials throughout the Neolithic in the North-West Mediterranean. Journal of Archaeological Science 126, 105309, doi: 10.1016/j.jas.2020.105309 (2021).

### Hjulström, B., Isaksson, S. & Hennius, A. organic geochemical evidence for pine tar production in middle Eastern Sweden during the Roman Iron Age. Journal of Archaeological Science 33, 283-294 (2006).

### 
