# Supplementary material for: Multiproxy study of 7500-year-old wooden sickles from the Lakeshore Village of La Marmotta, Italy
Source: Sci Rep. 2022 Sep 2;12:14976. doi: 10.1038/s41598-022-18597-8 (PMC9440057; doi:10.1038/s41598-022-18597-8)
Supplement: Supplementary file 5 — Supplementary Information 5. [file 41598_2022_18597_MOESM5_ESM.docx]

# S5. Phytolith results.

| **Sample n.** | **Phytoliths**  **1 g of sample** | **Monocotyledonous**  **(%)** | **Dicotyledonous wood/ bark (%)** | **Dicotyledonous leaves**  **(%)** | **Multicelled (articulated) phytoliths (%)** | **Weathered phytoliths (%)** |
| --- | --- | --- | --- | --- | --- | --- |
| 187878-7-1 | 330,000 | 14,2 | 71,1 | 5 | 17,7 | 9,7 |
| 187878-7-2 | 470,000 | 79,3 | 10,1 | 3,1 | 1,2 | 7,4 |
| 187879-6b-1 | 210,000 | 16,1 | 71,3 | 2,9 | 5,9 | 9,8 |
| 187879-6b-2 | 130,000 | 61,5 | 17,2 | 8,9 | 16,9 | 12,3 |
| 187882-12b-1 | 84,000 | 41,8 | 37,8 | 2,2 | 4,5 | 18,2 |
| 187882-12b-2 | 90,000 | 65,1 | 6,7 | 7,7 | 2,4 | 20,5 |

Supplementary Table S5: Main phytolith results obtained from adhesive materials.
