# Supplementary material for: Multiproxy study of 7500-year-old wooden sickles from the Lakeshore Village of La Marmotta, Italy
Source: Sci Rep. 2022 Sep 2;12:14976. doi: 10.1038/s41598-022-18597-8 (PMC9440057; doi:10.1038/s41598-022-18597-8)
Supplement: Supplementary file 6 — Supplementary Information 6. [file 41598_2022_18597_MOESM6_ESM.docx]

# S6. The flaked stone assemblage of La Marmotta and the sickle blades

La Marmotta lithic assemblage amounts to 12,402 items, of which: 10,964 on chert, 834 on obsidian, and 604 items on other indeterminate raw materials. It represents one of the largest assemblages for the Mediterranean Early Neolithic. Most chert correspond to local-regional raw materials of Apenninian provenance^1^. A small proportion is probably coming from the Gargano flint outcrops^2^. On the basis of preliminary analysis from a large sample of materials, obsidian is coming from the island of Palmarola, and, in a lesser extent, from the island of Lipari^3^.

Sickles inserts amount to 492 items, corresponding to the 4,5% of the total flaked stone assemblage on chert. This proportion is very similar to what observed in other Impressed Ware sites of the Central and Western Mediterranean^4^. Inserts are usually made on fragments of blade; only the 4.5% of them are on flake. The 72% of inserts is retouched: most of them, the 80% of retouched inserts, are truncations, single (62%) or double truncations (18%). Many truncations are also associated to lateral retouch and/or pseudo-retouch. The remaining are side-scrapers (16%), geometric backed tools (2,5%), borers (1%) and other indeterminate tools (0,5%). Averagely, inserts are 29-20 mm long, 13-10 mm wide, 3-2 mm thick.


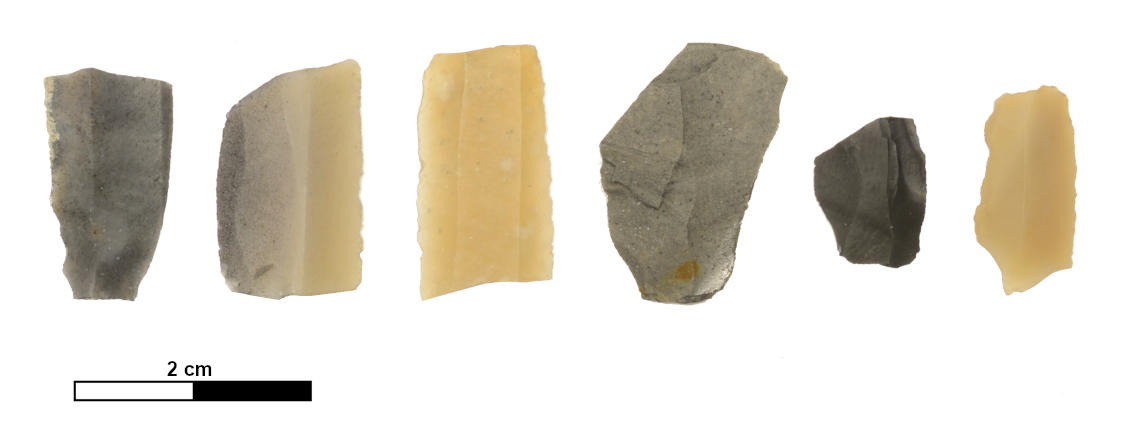


Figure S6-1. Stone inserts used for cereal harvesting from the flaked stone assemblage of La Marmotta.

# References

1. Radi, G. & Danese, E. L’abitato di Colle Santo Stefano di Ortucchio (L’Aquila). in Atti della XXXVI Riunione Scientifica IIPP, Preistoria e Protostoria dell’Abruzzo, Chieti-Celano, 27-30 settembre 2001, 145-161 (IIPP; Firenze, 2003).
2. Muntoni, I. M., Delluniversità, E., Allegretta, I., Terzano, R., & Eramo, G. Chert sources and Early to Middle Neolithic exploitation in the Tavoliere (Northern Apulia, Italy). Quaternary International, 615(30): 43-65 (2022). doi.org/10.1016/j.quaint.2021.01.016
3. De Francesco, A. M., Bocci, M., Crisci, G. M., & Francaviglia, V. Obsidian provenance at several Italian and Corsican archaeological sites using the non-destructive X-ray fluorescence method. in Obsidian and ancient manufactured glass (eds. Liritzis, I., & Stevenson, C. M.) 115-129 (Albuquerque, UNM Press, 2012).
4. Mazzucco, N., Ibáñez, J. J., Capuzzo, G., Gassin, B., Mineo, M., & Gibaja, J. F. Migration, adaptation, innovation: The spread of Neolithic harvesting technologies in the Mediterranean. PloS one, 15(4): e0232455 (2020) doi.org/10.1371/journal.pone.0232455
