# Supplementary material for: Multiproxy study of 7500-year-old wooden sickles from the Lakeshore Village of La Marmotta, Italy
Source: Sci Rep. 2022 Sep 2;12:14976. doi: 10.1038/s41598-022-18597-8 (PMC9440057; doi:10.1038/s41598-022-18597-8)
Supplement: Supplementary file 7 — Supplementary Information 7. [file 41598_2022_18597_MOESM7_ESM.docx]

# S7. La Marmotta macroremains (seeds and fruits, wood and charcoal): list of taxa (preliminary data)

| *Acer* sp.  *Agrimonia eupatoria* |
| --- |
| *Alchemilla* sp. |
| *Alnus glutinosa*  *Arenaria serpillifolia* |
| *Avena* sp. |
| cf. *Beta vulgaris* |
| *Brassica rapa* |
| *Bromus* cf. *secalinus* |
| *Capsella bursa-pastoris* |
| *Carex* cf. *pseudocyperus*  *Carex divulsa* type |
| *Carex* *elata-*type  *Carex remota-*type |
| *Carpinus* sp.  *Carthamus lanatus* |
| *Cerastium fontanum* |
| *Ceratophyllum demersum* |
| *Chara* sp. pl. |
| *Chenopodium album* |
| *Cladium mariscus* |
| *Cornus mas* |
| *Cornus sanguinea* |
| *Corylus avellana* |
| *Cyperus* cf. *longus* |
| *Cyperus fuscus/flavescens*  *Dianthus* sp. |
| *Echinochloa crus-galli* |
| *Eleocharis* cf. *palustris* |
| *Eleocharis uniglumis* |
| *Euphorbia* cf. *helioscopia* |
| *Fallopia convolvulus* |
| *Ficus carica* |
| *Fragaria vesca/viridis* |
| *Fraxinus* spp. |
| *Fumaria officinalis* |
| *Heliotropium europaeum* |
| *Hordeum distichum* |
| *Hypericum* *perforatum* |
| *Juncus* spp. |
| *Lathyrus cicera/sativus* |
| *Lathyrus* *occidentalis-*type |
| *Laurus nobilis* |
| *Lens culinaris* |
| *Linum* cf. *bienne* |
| *Linum strictum*  *Linum usitatissimum* |
| *Lycopus europaeus* |
| *Lychnis flos-cuculi*  *Malus sylvestris* |
| *Malva nicaeensis/sylvestris* |
| *Medicago* cf. *arabica* |
| *Medicago lupulina* |
| *Medicago* cf.  *minima*  *Mentha* spp. |
| *Myriophyllum alternifolium* |
| *Myriophyllum verticillatum* |
| *Najas flexilis* |
| *Najas marina* |
| *Najas minor* |
| *Nitella* sp. pl. |
| *Nuphar lutea* |
| *Ornithopus* sp. |
| *Ostrya carpinifolia* |
| *Papaver somniferum* |
| *Petrorhagia* cf. *prolifera* |
| *Phragmites australis* |
| *Physalis alkekengi* |
| *Picris hieracioides* |
| *Pisum sativum* |
| *Plantago* cf. *media* |
| *Poa* sp. |
| *Polygonum aviculare* |
| *Persicaria hydropiper/minor* |
| *Persicaria lapathifolia* |
| *Persicaria maculosa* |
| *Populus* sp. |
| *Portulaca oleracea* |
| *Potamogeton* sp. pl. |
| *Potentilla reptans* |
| *Prunus domestica* subsp. *insititia* |
| *Prunus spinosa* |
| *Pteridium aquilinum* |
| *Pyrus* sp. |
| *Quercus* cf. *ilex* |
| *Quercus* sez. *Robur* |
| *Ranunculus repens* |
| *Ranunculus sardous* |
| *Ranunculus sceleratus* |
| *Ranunculus* sect. *Batrachium* |
| *Rosa* sp.  Rosaceae/Maloideae |
| *Rubus* cf. *caesius* |
| *Rubus fruticosus* agg. |
| *Rubus idaeus* |
| *Rumex* cf. *crispus* |
| *Rumex sanguineus/conglomeratus* |
| *Sambucus ebulus* |
| *Sambucus nigra/racemosa*  *Scabiosa* sp. |
| *Schoenoplectus lacustris/tabernaemontani* |
| *Setaria verticillata/viridis* |
| *Silene alba/dioica* |
| *Silene cretica* |
| *Silene gallica* |
| *Silene vulgaris* |
| *Silybum marianum*  *Solanum nigrum* |
| *Sparganium* sp. |
| *Spergularia* sp. |
| *Stellaria media* |
| *Trifolium* sp. |
| *Triticum durum/turgidum* |
| *Triticum dicoccum* |
| *Triticum monococcum* |
| *Typha* sp. |
| *Urtica dioica* |
| *Valerianella dentata* |
| *Verbena officinalis* |
| *Veronica* cf. *anagallis-aquatica* |
| *Viburnum lantana* |
| *Viburnum tinus* |
| *Vicia* cf. *faba* |
| *Vicia* cf. *sativa* |
| *Vicia* cf. *ervilia* |
| *Viola riviniana/reichenbachiana* |
| *Vitis vinifera sylvestris* |
