# Supplementary material for: Multiproxy study of 7500-year-old wooden sickles from the Lakeshore Village of La Marmotta, Italy
Source: Sci Rep. 2022 Sep 2;12:14976. doi: 10.1038/s41598-022-18597-8 (PMC9440057; doi:10.1038/s41598-022-18597-8)

ARTEFACT 44297 (SICKLE)

Capture: Breuckmann Smartscan3D duo.

3D model reconstruction: Rapidform/Geomagic+Meshlab.

3D-PDF: Adobe Acrobat X Pro.

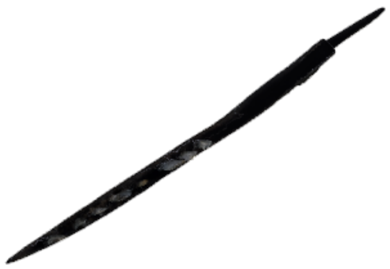

Supplement: Supplementary file 10 — Supplementary Information 10. [file 41598_2022_18597_MOESM10_ESM.pdf]
